# Antimicrobial Activity of Rhenium Di- and Tricarbonyl Diimine Complexes: Insights on Membrane-Bound *S. aureus* Protein Binding

**DOI:** 10.3390/ph15091107

**Published:** 2022-09-05

**Authors:** Kevin Schindler, Youri Cortat, Miroslava Nedyalkova, Aurelien Crochet, Marco Lattuada, Aleksandar Pavic, Fabio Zobi

**Affiliations:** 1 Department of Chemistry, Fribourg University, Chemin Du Musée 9, 1700 Fribourg, Switzerland; 2 Institute of Molecular Genetics and Genetic Engineering, University of Belgrade, Vojvode Stepe 444a, 11042 Belgrade, Serbia

**Keywords:** rhenium, tricarbonyl, antimicrobial, *S. aureus*, MRSA, AutoDock, membrane, proteins, LspA, LtaA

## Abstract

Antimicrobial resistance is one of the major human health threats, with significant impacts on the global economy. Antibiotics are becoming increasingly ineffective as drug-resistance spreads, imposing an urgent need for new and innovative antimicrobial agents. Metal complexes are an untapped source of antimicrobial potential. Rhenium complexes, amongst others, are particularly attractive due to their low in vivo toxicity and high antimicrobial activity, but little is known about their targets and mechanism of action. In this study, a series of rhenium di- and tricarbonyl diimine complexes were prepared and evaluated for their antimicrobial potential against eight different microorganisms comprising Gram-negative and -positive bacteria. Our data showed that none of the Re dicarbonyl or neutral tricarbonyl species have either bactericidal or bacteriostatic potential. In order to identify possible targets of the molecules, and thus possibly understand the observed differences in the antimicrobial efficacy of the molecules, we computationally evaluated the binding affinity of active and inactive complexes against structurally characterized membrane-bound *S. aureus* proteins. The computational analysis indicates two possible major targets for this class of compounds, namely lipoteichoic acids flippase (LtaA) and lipoprotein signal peptidase II (LspA). Our results, consistent with the published in vitro studies, will be useful for the future design of rhenium tricarbonyl diimine-based antibiotics.

## 1. Introduction

The expansion of resistance to conventional antibiotics has become a notable health threat and necessitated the development of alternative treatment options for battling such a global problem [1]. Amongst the six nosocomial pathogens that exhibit multidrug resistance and virulence, the methicillin-resistant *S. aureus* (MRSA) is a major cause of community- and hospital-acquired infections worldwide, ranging from superficial skin and soft tissue infections [2] to invasive infections and sepsis [3]. This pathogen represents the most common and the second most common cause of healthcare-associated and bloodstream infections (BSI), as well as the most important cause of BSI death [4]. Since the bacterium is increasingly showing resistance to multiple antibiotics, in 2017 the World Health Organization listed it among the high priority group of human pathogens. Indeed, the same year, the Centers for Disease Control and Prevention (CDC) reported that more than 119,000 people suffered from *S. aureus* bloodstream infections in the United States, with nearly 20,000 of them (>16%) eventually dying.

What has exacerbated the problem of antimicrobial resistance (AMR) is the fact that fewer new antibiotics are reaching the market, with the last entirely original class of antibiotics discovered in the late 1980s. This is because large pharmaceutical companies have left the market due to a lack of financial incentives [5]. Consequently, in the last few years, academic research groups at universities around the globe have taken up the challenge to prepare and discover new antibiotic drugs that may serve as lead compounds for new structurally viable drugs. In our era, strategies for the discovery and development of new drugs combine computational and experimental approaches. This is true in virtually all medicinal discovery areas, including the design and discovery of molecules as appropriate candidates for treatment of *Staphylococcus aureus* infection. Computer-aided drug design (CADD) methods are computational approaches to guiding and expediting the experimental findings for new drug design processes [6,7,8,9,10]. CADD can be used in a qualitative and quantitative mode to improve the biovalidity and prediction rates for ligand binding affinity, as well as specificity, in a manner that can lead to the identification of biological targets of known drugs and the design process of new agents in a simpler, more efficient, and less expensive manner. In a modern drug design process, typically hundreds of compounds can be tested in a short time. The existing methodologies such as, e.g., site-identification by ligand competitive saturation (SILCS) [8,11], have become versatile tools for ligand–protein binding prediction. The foundation of the CADD technique is based on molecular docking [12,13,14] and molecular dynamics simulations [15,16].

Within the specific context of this article, CADD has been used, e.g., to evaluate medicinal plant-derived active compounds that could be used as therapeutic alternatives for MRSA infection [17,18,19,20]. The study of receptor–ligand interactions in the framework of molecular docking has drawn attention to the importance of probing the efficiency of these plant-derived antimicrobial agents [17,18] and testing antimicrobial activity using screened lead compounds, focusing on the role of computational screening methods [20,21,22,23] in tackling the problem. However, a major strategy still pursued in the field is that of modifying already approved antibiotics [24]. As may be expected, all of these molecules are purely organic compounds. While some of these new derivatives (some are currently in preclinical or clinical development) will provide feasible short-term solutions, it is probable that the pathogens will rapidly adapt and develop resistance to these molecules as well [24].

As an alternative to organic compounds, there is an increasing awareness in academia of the potential of metal complexes to act as the new class of molecules for this purpose. Indeed, the unique chemistry and larger variety of 3D geometries of metal compounds can address targets and modes of action that are unavailable to organic molecules. Over the last decade, complexes of virtually all the transition metals have been evaluated as antimicrobial agents [25,26,27], with rhenium (Re), among others [28,29], showing promising potential for new antibiotic development [30,31,32,33]. While other transition metal complexes [34,35,36,37,38,39,40,41,42,43,44,45,46,47] act against Gram-negative bacteria, carbonyl rhenium complexes have demonstrated a very potent activity towards Gram-positive pathogens, particularly towards *Staphylococcus aureus*, involving both the methicillin-resistant (MRSA) and methicillin-sensitive (MSSA) strains [31,32,33,48,49,50,51].

Our group is principally interested in the development of the chemistry of carbonyl rhenium complexes for their use in different medicinal applications, including their evaluation as anticancer [52,53,54] and antibacterial agents [49,50]. Due to their very low in vivo toxicity [55,56,57,58], tricarbonyl complexes of rhenium are particularly attractive. The same type of molecules (i.e., those of the *fac*-[Re(CO)_3_]^+^ core) are also the ones most widely investigated, showing the highest anticancer and antimicrobial effectiveness against *S. aureus* strains [59]. It is still unclear what molecular features of carbonyl rhenium complexes make them such promising medicinal agents. In a study that we have recently reported [49], we concluded that, unlike anticancer complexes, positively charged rhenium species are most effective against the microbes, and we hypothesized that charged compounds may interact more effectively with phosphatidylglycerol and cardiolipin anionic membrane lipids. Later, however, we found that, by substituting a single neutral carbonyl with a nitrosonium cation, the compounds lose their antimicrobial effect [60]. Thus, in terms of their antibacterial effectiveness, both the required molecular features and mechanism of action of these agents remain largely unknown.

In order to advance knowledge of the issues mentioned above, we evaluated the antimicrobial activity of dicarbonyl rhenium diimine complexes (i.e., of the *cis*-[Re(CO)_2_]^+/2+^ core) and compared it to that of structurally similar *fac*-[Re(CO)_3_]^+^ species. This part of study was performed because (a) no antimicrobial data is available on the carbonyl complexes of the *cis*-[Re(CO)_2_]^+/2+^ core lacking other π-acid ligands, and (b) a comparison of the activity of *cis*-[Re(CO)_2_]^+/2+^ and structurally similar *fac*-[Re(CO)_3_]^+^ species may provide information about the key molecular features required for the design of an effective Re-based antibiotic agent. Furthermore, we computationally evaluated the binding affinity of all the compounds (both active and inactive molecules) against structurally characterized membrane-bound *S. aureus* proteins. We performed this study principally to (a) identify possible biological targets of the active complexes; (b) possibly understand the underlying reasons for the observed differences in the antimicrobial efficacy of Re complexes; and (c) offer support for the rational design of rhenium complexes based on the computational protocol for CADD.

## 2. Results and Discussion

### 2.1. Synthesis and Characterization of the Metal Complexes

**Scheme 1:**
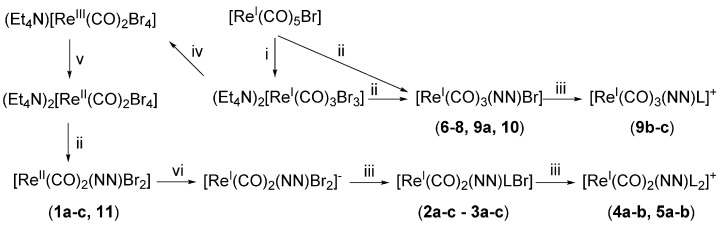
Synthetic scheme for the preparation of the complexes investigated in this study. NN = relevant bidentate diamine ligand; L = pyridine (py) or *N*-methyl imidazole (MeIm). General conditions: i: Et_4_NBr, diglyme; ii: NN, ethanol/water, toluene, or CH_2_Cl_2_; iii: L, methanol or neat L; iv: Br_2_, CH_2_Cl_2_; v: tetrakis(dimethylamino)ethylene, acetonitrile, under N_2_; vi: tetrakis(dimethylamino)ethylene, CH_2_Cl_2_, under N_2_. For more details, refer to Section 4.

**Figure 1:**
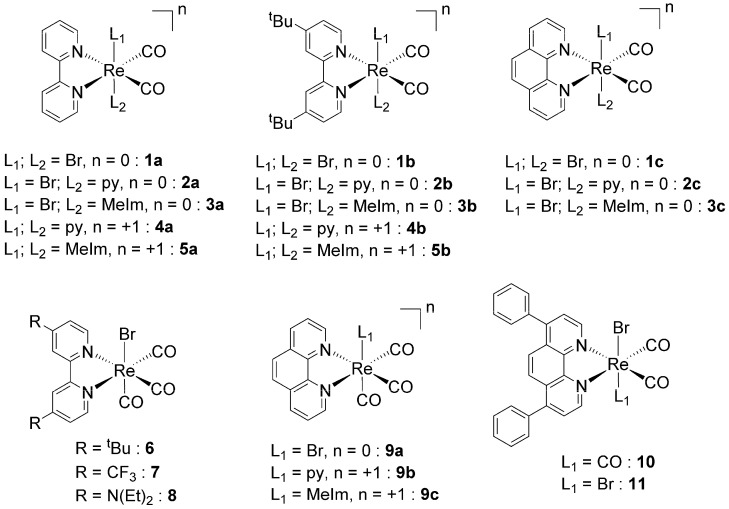
Structures and codes of the tested Re dicarbonyl (**1**–**5**) and tricarbonyl (**6**–**10**) complexes.

Rhenium carbonyl complexes investigated in this study were prepared according to the procedures illustrated in Scheme 1. Tricarbonyl species **6**–**10** (Figure 1) were obtained in a high yield and purity, according to established routes generally used in the preparation of these compounds. The *fac*-[Re(CO)_3_(NN)Br] complexes (**6**–**8**, **9a**, and **10**, where NN = relevant bidentate diimine ligand) may be obtained in one step from [Re(CO)_5_Br] by boiling this precursor in toluene in the presence of one equivalent of NN. The resulting yellow product, isolated by filtration, is generally of a high purity (>96% by NMR or HPLC) and can be used for further modification by the substitution reaction of the coordinated bromide atom by other monodentate ligands, as in the case of species **9b** and **9c**. For this reaction, we found that the best conditions consist in the treatment of a *fac*-[Re(CO)_3_(NN)Br] complex with trifluoromethanesulfonic acid to produce the intermediate *fac*-[Re(CO)_3_(NN)(CF_3_SO_3_)] molecule, followed by the addition of L (where L = pyridine: py or *N*-methyl imidazole: MeIm). The reaction is also high yielding, but the desired *fac*-[Re(CO)_3_(NN)L]CF_3_SO_3_ salt requires purification on alumina or via HPLC.

The preparation of dicarbonyl *cis*-[Re(CO)_2_(NN)X_2_] species (**1**–**5** and **11**, where X = Br or L, Figure 1) is more demanding and requires several steps, starting from the common [Re(CO)_5_Br] precursor. We have recently published the details of this chemistry [61], showing that the synthetic route is favorable if X is a halide or an aromatic heterocycle (or a combination of both). However, yields of *cis*-[Re(CO)_2_(NN)X_2_] species are much lower than the comparable *fac*-[Re(CO)_3_(NN)Br] complexes. Briefly, *cis*-[Re(CO)_2_(NN)X_2_] species may be prepared following the sequential two-electron oxidation of *fac*-[Re(CO)_3_Br_3_]^2−^ to *cis*-[Re(CO)_2_Br_4_]^−^ [62], its one-electron reduction to *cis*-[Re(CO)_2_Br_4_]^2−^, the complexation of NN to *cis*-[Re(CO)_2_(NN)Br_2_], its one-electron reduction to *cis*-[Re(CO)_2_(NN)Br_2_]^−^, and, finally, the stepwise substitution of Br with L to obtain *cis*-[Re(CO)_2_(NN)BrL_2_] and *cis*-[Re(CO)_2_(NN)L_2_]^+^. It is interesting to point out here that, contrary to other similar complexes, the presence of NN in the coordination sphere of the 17-electron Re^II^ complexes (**1a**–**c** and **11**) imparts stability to the molecules, which are stable in solution and do not decompose by releasing CO [63,64].

**Figure 2:**
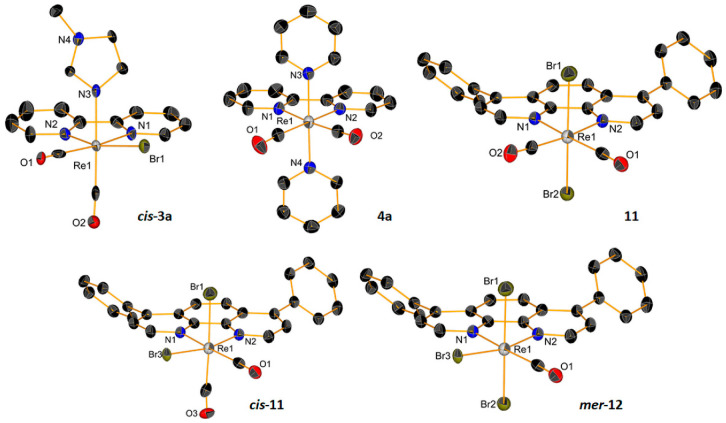
ORTEP representations of the crystal structures of Re dicarbonyl complexes. Thermal ellipsoids are at 30% probability. Hydrogen atoms are omitted for clarity. **Note**: the compounds *cis*-**11** and *mer*-**12** co-crystallize in a mixture, where **10** and **11** are also present.

**Figure 3:**
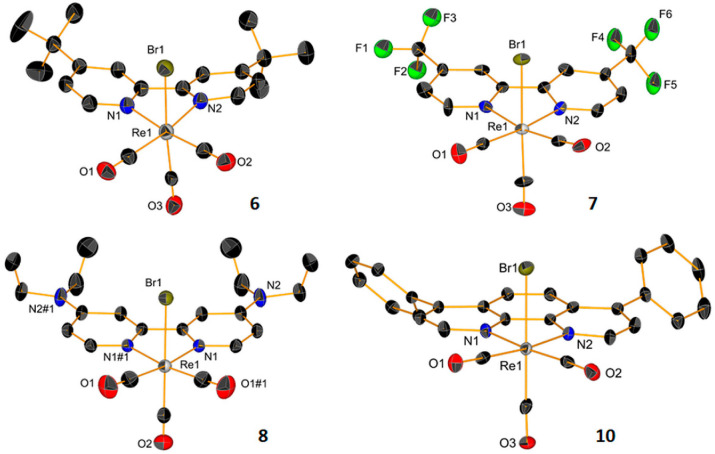
ORTEP representations of the crystal structures of Re tricarbonyl complexes **6**–**8** and **10**. Thermal ellipsoids are at 30% probability. Hydrogen atoms are omitted for clarity.

New complexes were characterized by standard techniques, including X-ray crystallography for the dicarbonyl species **4a** and **11** (Figure 2) and tricarbonyl complexes **6**–**8** and **10** (Figure 3, with relevant bond distances and angles for the solid-state molecular structures in the Supplementary Materials). Within the series of dicarbonyl *cis*-[Re(CO)_2_(NN)Br_2_] species, the preparation of compound **11** (where NN = bathophenanthroline: batho-phen) was particularly challenging. Indeed, the reaction of either *cis*-[Re^III^(CO)_2_Br_4_]^−^ or *cis*-[Re^II^(CO)_2_Br_4_]^2−^ with bathophen leads to a mixture of products which are very difficult to separate. Normally, *cis*-[Re(CO)_2_(NN)Br_2_] complexes are obtained as *cis*-*cis*-*trans* species (with the two Br atoms in the *trans* position to each other). Only when one of the bromides is substituted for L, the intermediate penta-coordinated complexes undergo Berry pseudorotation, which establishes an equilibrium between the *cis*-*cis*-*trans* and *cis*-*cis*-*cis* isomers [61]. These can be separated by column chromatography and crystallized separately (as in the case of **3a**, Figure 2). In the preparation of **11**, we found not only that the reaction leads to disproportionation, giving **10**, but also that the *cis*-*cis*-*cis* isomer of **11** (*cis*-**11**) and the mono carbonyl *mer*-[Re(CO)(NN)Br_3_] complex (*mer*-**12**, Figure 2) are formed. Complex **11** can be separated from the mixture, but despite our efforts, the other complexes formed could not be eluted separately in our chromatographic purification procedures. We should underline here that we were able to identify the products obtained in the reaction only by co-crystallizing them in a mixture. We also note that, to our knowledge, *mer*-[Re(CO)(NN)Br_3_] (*mer*-**12**) is a unique example of a diimine rhenium mono carbonyl complex that is structurally characterized.

### 2.2. Antimicrobial Properties of the Complexes

**Figure 4:**
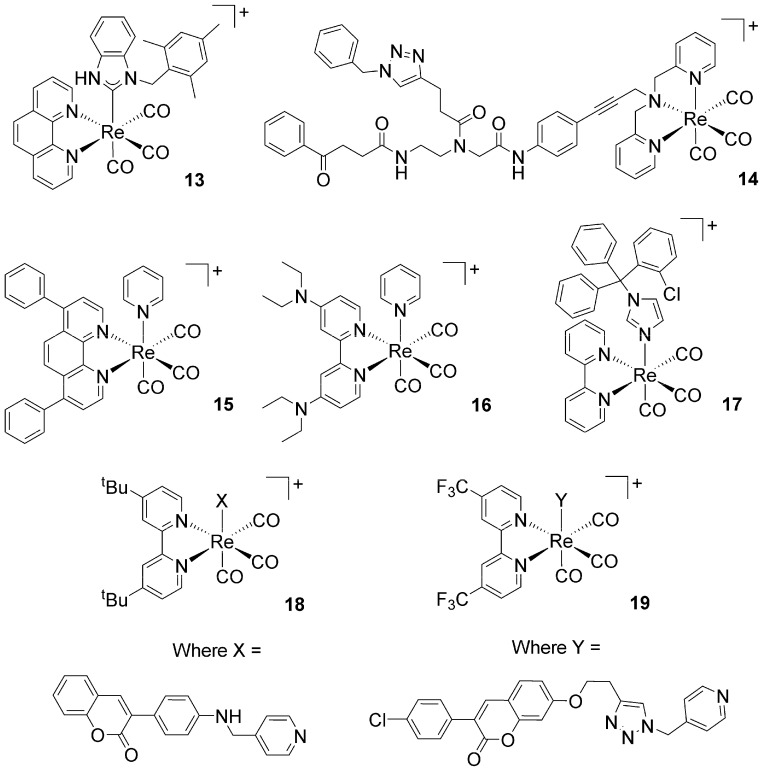
Structures of previously published active *fac*-[Re(CO)_3_]^+^ complexes. Complex **13** [32]; complex **14** [31]; complexes **15**, **16**, **18**, and **19** [49,50]; complex **17** [48].

The antimicrobial activity of complexes **1**–**11** (15 neutral, 6 cationic) was determined against eight different microorganisms, including four Gram-negative bacteria (*Enterobacter cloacae* ATCC 3047, *Klebsiella pneumoniae* ATCC 13803, *Acinetobacter baumannii* ATCC 19606, and *Pseudomonas aeruginosa* PAO1 NCTC10332), two Gram-positive bacteria (methicillin-resistant *Staphylococcus aureus* MRSA43300 and methicillin-sensitive *S. aureus* ATCC25923), and two fungi (*Candida albicans* SC5314 and *C. auris*, a clinical strain). The species of these two genera are responsible for the majority of hospital-acquired infections and are challenging to treat, especially in the case of their co-infections [65]. The results of our study are given in Table 1. We found that none of the dicarbonyl complexes showed antimicrobial potential. Only compounds **4b**, **5b**, and **11** were weakly active against the *S. aureus* strains, but their MIC values (25 and 50 μM, respectively) were much higher than those of the active rhenium complexes **13**–**19** (Figure 4) [31,32,48,49,50].

### 2.3. Molecular Docking Study: Membrane-Bound S. aureus Proteins

The results obtained from our in vitro antimicrobial investigation raised a fundamental question, namely: “what sets apart cationic *fac*-[Re(CO)_3_]^+^ complexes from other structurally similar neutral complexes or compounds lacking the tricarbonyl core?” Or, in other words, “why are complexes **13**–**19** (Figure 4) active antimicrobial agents while other rhenium complexes are not?” Compounds **13**–**19** are different molecules, but they share some common features (e.g., the same charge, with a lipophilic diimine or polydentate ligand with a pyridine in the coordination sphere). In addition, Table 2 presents the predictability rates of the drug-likeness properties for these rhenium complexes. The descriptor values were retrieved using the AlvaDesc v.2 software (Milano, Italy) [66].

At this early stage of investigation, to aid in our search for an answer to the question, a purely experimental approach focused on, e.g., microbial gene expression analysis and transcriptomic data would be costly and time consuming. We thus decided to adopt an in silico approach in order to guide future synthetic, SAR, and mechanistic studies. There are, fortunately, some experimental facts that helped us to focus our attention on specific enzymes that may be considered as possible targets for one or more of the compounds **13–19**. Although mechanistic studies are limited and specific biological targets are still unknown, the effective antimicrobial *fac*-[Re(CO)_3_]^+^ complexes appear to act predominately on the membrane of the bacteria. The complex of Metzler-Nolte and Bandow, i.e., compound **14** in Figure 4, targets the cytoplasmic membrane of *Bacillus subtilis*, affecting its architecture and disrupting essential cellular processes taking place at the membrane, such as respiration, as well as cell wall biosynthesis and integrity [30]. Similarly, Mendes et al. have shown that the mechanism of action of the *fac*-[Re(CO)_3_(bpy)(ctz)]^+^ complex (**17** in Figure 4, where ctz = the drug clotrimazole) involves a sequence of events initiated by membrane insertion, followed by membrane disorganization, the inhibition of peptidoglycan biosynthesis, and the breakdown of the membrane potential [48].

Based on these data, in order to possibly understand the differences in the antimicrobial effects of the previously published active *fac*-[Re(CO)_3_]^+^ complexes (**13**- **19**, Figure 4) and inactive *fac*-[Re(CO)_3_]^+^ and *cis*-[Re(CO)_2_]^n^ complexes, we decided to investigate the binding affinity of all the above compounds against membrane-bound *S. aureus* proteins. The in silico docking studies were also performed in order to gain insights on the possible targets of the molecules by the careful analysis of the data. A PDB search revealed that nine structurally characterized membrane-bound *S. aureus* MRSA proteins are available on the database. Of these, we selected eight, comprising four penicillin-binding proteins (PBPs) [67,68,69,70] and the following enzymes: lipoteichoic acid synthase [71] (specifically its extracellular catalytic domain, eLtaS), type-I signal peptidase (SpsB) [72], lipoprotein signal peptidase II (LspA) [73], and lipoteichoic acid flippase (LtaA) [74]. The pre-screening of the binding affinities (*b.a.*) was performed with the AutoDock Vina software [14]. The calculated *b.a.* were recorded as docking scores in kilocalories per mole (kcal/mol), and the results are given in the Supplementary Materials (Table S1a,b). In the initial screening, the metal complexes were first docked at the known inhibitor-binding site of the specific protein, and the *b.a.* was compared to that of the same inhibitor. At this stage, only proteins where complexes showed a *b.a.* of ca. −9.0 kcal/mol that was greater than the corresponding inhibitor’s *b.a.* (Δ values in Table S1a,b), or a *b.a.* of ca. −10.0 kcal/mol that was comparable to the corresponding inhibitor’s *b.a.,* were considered as possible targets for the complexes.

Within the abovementioned constrains, in general terms, our initial analysis revealed the following (detailed values are in the Supplementary Materials, Table S1a,b):

(1) With the exception of the *cis*-[Re(CO)_2_]^n^ complexes **1b**–**3b** and the *fac*-[Re(CO)_3_]^+^ complexes **6**, **7**, and **10**, none of the inactive rhenium di- or tricarbonyl compounds showed any *b.a.* for the enzyme evaluated.
(2) The inactive molecules **1b**–**3b**, **6**, **7**, and **10** showed an affinity for the penicillin-binding protein 4 (PBP4), with *b.a.* values ranging from −8.9 (**1b**) to −12.3 (**10**) kcal/mol.
(3) Compound **10** also showed a strong affinity for lipoteichoic acid flippase (LtaA), with a *b.a.* of −10.3 kcal/mol.
(4) Amongst the active antimicrobial rhenium complexes (i.e., molecules **13**–**19**, Figure 4), complexes **16** and **17** showed the lowest *b.a.* values for the selected enzymes. These were higher than those of the inactive compounds but lower than those of the known inhibitors.
(5) With variations within the series, the other active antimicrobial rhenium complexes (**13**–**15** and **18**–**19**) showed good *b.a.* values for five enzymes. These were the penicillin-binding protein 4 (PBP4, *b.a.* ranging from −9.1 (**13**) to −10.7 (**19**) kcal/mol); type-I signal peptidase (SpsB, all complexes except **14**, *b.a.* ranging from −9.1 (**13**) to −10.4 (**19**) kcal/mol); lipoteichoic acid synthase (LtaS, only **15**, **18**, and **19**, *b.a.* ranging from −9.4 (**15**) to −10.9 (**19**) kcal/mol); lipoteichoic acid flippase (LtaA, only **15**, **18**, and **19**, *b.a.* ranging from −10.4 (**15**) to −11.3 (**19**) kcal/mol).; and lipoprotein signal peptidase II (LspA, all complexes except **13**, *b.a.* ranging from −8.7 (**15**) to −10.6 (**18**) kcal/mol).

Interestingly, PBP4, LtaS, and LtaA are all involved in bacterial wall biosynthesis [70,72,74,75,76,77,78]. PBP4 is a transpeptidase that performs the crosslinking reaction in the synthesis of the peptidoglycan backbone [78]. LtaS catalyzes the polymerization of lipoteichoic acid (LTA) polyglycerol phosphate, a reaction that presumably uses phosphatidylglycerol as a substrate [71]. This enzyme is required for staphylococcal growth and the cell division process [79,80]. LtaA acts upstream of LtaS [81], and it is presumed to catalyze the translocation reaction of anchor lipid-linked disaccharide gemfibrozil-diacylglycerol from the cytoplasmic leaflet of the membrane to the extracellular side of the plasma membrane, where lipoteichoic acids are assembled [74,75,76,77]. A flippase with a similar structure (MurJ) [82,83] is also involved in the translocation of disaccharide-pentapeptide building blocks, attached to a polyisoprene lipid carrier (called lipid II), across the cytoplasmic membrane, where peptidoglycan polymerization (i.e., the polysaccharide matrix that protects bacteria from osmotic lysis) takes place [77]. The remaining two proteins are SpsB and LspA. SpsB is a proteolytic enzyme that plays a crucial role in bacterial viability by processing proteins that are translocated across the membrane [72,84], while LspA is involved in bacterial lipoprotein post-translational processing [85] and is essential for the survival and virulence of Gram-positive bacteria [86,87]. This latter enzyme is considered as one of the major targets for the development of new antibiotics [88]. The calculated binding affinities of active Re complexes for these possible targets (*b.a.* ranging from ca. −9 to −11 kcal/mol) are fully consistent with the experimental results reported by Wenzel et al. [30] and Mendes et al. [48], in that the inhibition of these proteins leads to membrane disorganization and affects peptidoglycan/wall biosynthesis [70,74,75,76,77,78].

Following this initial screening, the active complexes **13–19** were more comprehensively analyzed for their binding affinities for the selected receptors. Extensive semi-flexible docking was performed, introducing flexibility into the amino acid side chains and complex rotatable bonds of the binding pockets of the receptors. The number of modes was set to 200, and the exhaustiveness was set to 40. Each docked complex was calculated in triplicate mode. The triplication test detects whether there is a variation in the obtained cluster compactness of the poses and changes in the top-ranked compounds from the previous run; thus, one avoids bias in the scoring. If a bias in the scoring is present, the solution for such a case, along with the control experiment, is a reduction in the chemical space search (e.g., narrowing of the search box). The performed protocol provides information as to whether the best selected molecules remain amongst the highest scored compounds in the rank-ordered docking list. After the calibration procedure for the docking, the molecules were virtually screened against the eight target proteins. The localization of the active pocket amino acid residues was predicted according to Jendele et al. [89]. The results are summarized in Table 3, while the detailed ranking of the obtained pockets is in the Supplementary Materials (Table S2).

Accordingly, the computational results of this library of compounds are shown in Table 4. For the PBP receptors, the docking protocol identified **15** and **19** as having the greatest *b.a.* for these enzymes, particularly for PBP2a and PBP4 (Table 4). As other non-active rhenium complexes showed a *b.a.* for PBP4, we posit that this protein is not a probable target for active complexes. Conversely, the *b.a.* of **15** and **19** for PBP2a is of interest (*b.a.* values of −9.2 and −9.8 kcal/mol respectively, Table 4). The expression of the penicillin-binding protein 2a (PBP2a) is responsible in methicillin-resistant *S. aureus* (MRSA) for the high-level resistance of the bacteria to β-lactam antibiotics [78]. PBP2a is a unique transpeptidase, as it is capable of catalyzing cell wall crosslinking despite the presence of β-lactam antibiotics. The inhibition of PBP2a by **15** and **19** may thus possibly additionally account for the strong antimicrobial activity of these complexes against MRSA [49,50]. Computationally, in the case of **19**, the stabilization of the protein–drug complex is based on the detected H-bonds between the compound and the surrounding amino acid environment (Ser, Thr, and Gln residues). A detailed distribution of the amino acids of the best complexes is given in the Supplementary Materials (Table S3). In this case, the intramolecular backbone H-bonds stabilize the β-turn structure with the ligand position.

**Figure 5:**
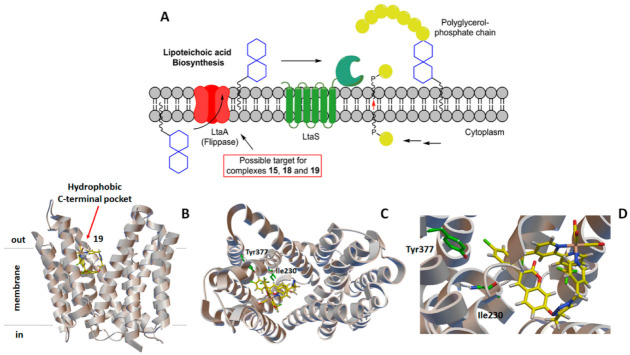
(**A**) Schematic diagram of the lipoteichoic acid synthetic machinery in MRSA with the possible target of the active antimicrobial rhenium complexes. For more details about scheme (**A**), see [75]. Computer-generated lowest energy pose of the selected complex **19** in the hydrophobic C-terminal pocket of lipoteichoic acid flippase (LtaA): (**B**) side view; (**C**) top view; (**D**) detail of the binding region. In (**C**,**D**), the two amino acid residues most likely involved in the H-bonding interactions with **19** are shown in green.

**Figure 6:**
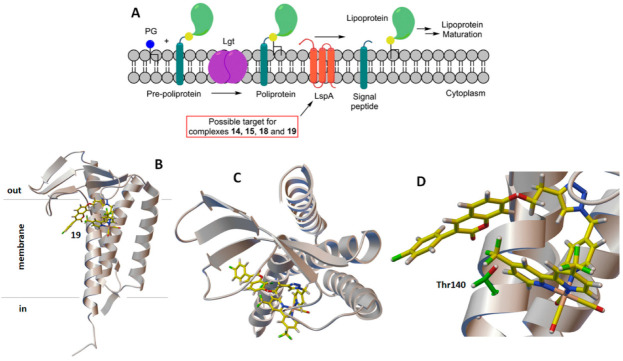
(**A**) Schematic diagram of the lipoprotein post-translational processing pathway with the possible target of the active antimicrobial rhenium complexes. For more details about scheme (**A**), see [73]. Computer-generated lowest energy pose of selected complex **19** in lipoprotein signal peptidase II (LspA): (**B**) side view; (**C**) top view; (**D**) detail of the binding region. In (**C**,**D**), the amino acid residue most likely involved in the H-bonding interactions with **19** is shown in green.

For the second group of receptors (namely LtaS, SpsB, LtaA, and LspA), the docking protocol identified complexes **14**, **15**, **18**, and **19** as having a high *b.a.* for lipoteichoic acid flippase (LtaA, all complexes except **14**) and lipoprotein signal peptidase II (LspA, see Table 4). As mentioned above, flippases such as LtaA catalyze the translocation reactions of anchor lipid-linked disaccharide gentiobiosyl-diacylglycerol and lipid II across the cytoplasmic membrane, where essential cell wall polymers (i.e., lipoteichoic acid and peptidoglycan) are assembled (Figure 5) [74,75,76,77,81,82,83]. LspA, on the other hand, is involved in bacterial lipoprotein post-translational processing [85] and is essential for the survival and virulence of Gram-positive bacteria (Figure 6) [86,87]. The possible inhibition of these enzymes by active antibiotic rhenium complexes would disrupt essential cellular processes taking place at the membrane and ultimately lead to cell death. It should be mentioned that our computational analysis did not identify possible targets of complexes **13**, **16**, and **17**. If, for the former complexes, this indicates that the compounds may exert their antibiotic activity against MRSA via mechanisms not involving membrane-bound proteins, for **17**, the results appear to support the experimental evidence of Mendes et al. [48]. Indeed, the authors reported that **17** interferes with the cycling of the undecaprenyl precursor in peptidoglycan biosynthesis (“lipid II cycle”), leading to the accumulation of UDP-MurNAc-pentapeptide (i.e., lipid I, the ultimate cytoplasmic peptidoglycan precursor) in the cytoplasm of treated cells. Thus, **17** inhibits the MurG-mediated conversion of lipid I to lipid II [31]. The X-ray structure of MRSA MurG is not available in the PDB database; thus, we could not computationally confirm the experimental data of Mendes et al. [48].

**Figure 7:**
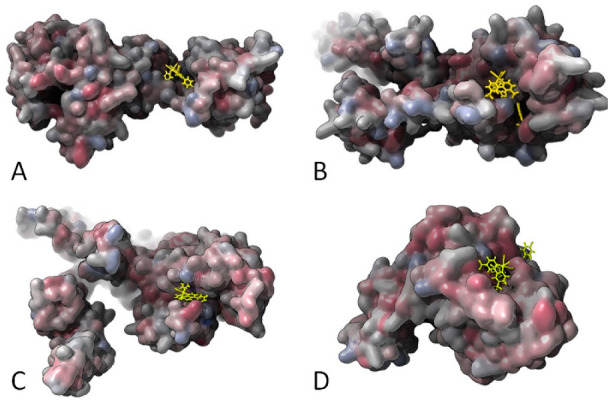
Gaussian surface representation of the hydrophobicity of (**A**) **15** and PBP2 (2OLV); (**B**) **19** and PBP2a (4DKI); (**C**) **15** and PBP3 (3VSL); and (**D**) **19** and PBP4 (5TXI). The red-blue color palette changes from hydrophilic blue to hydrophobic red.

**Figure 8:**
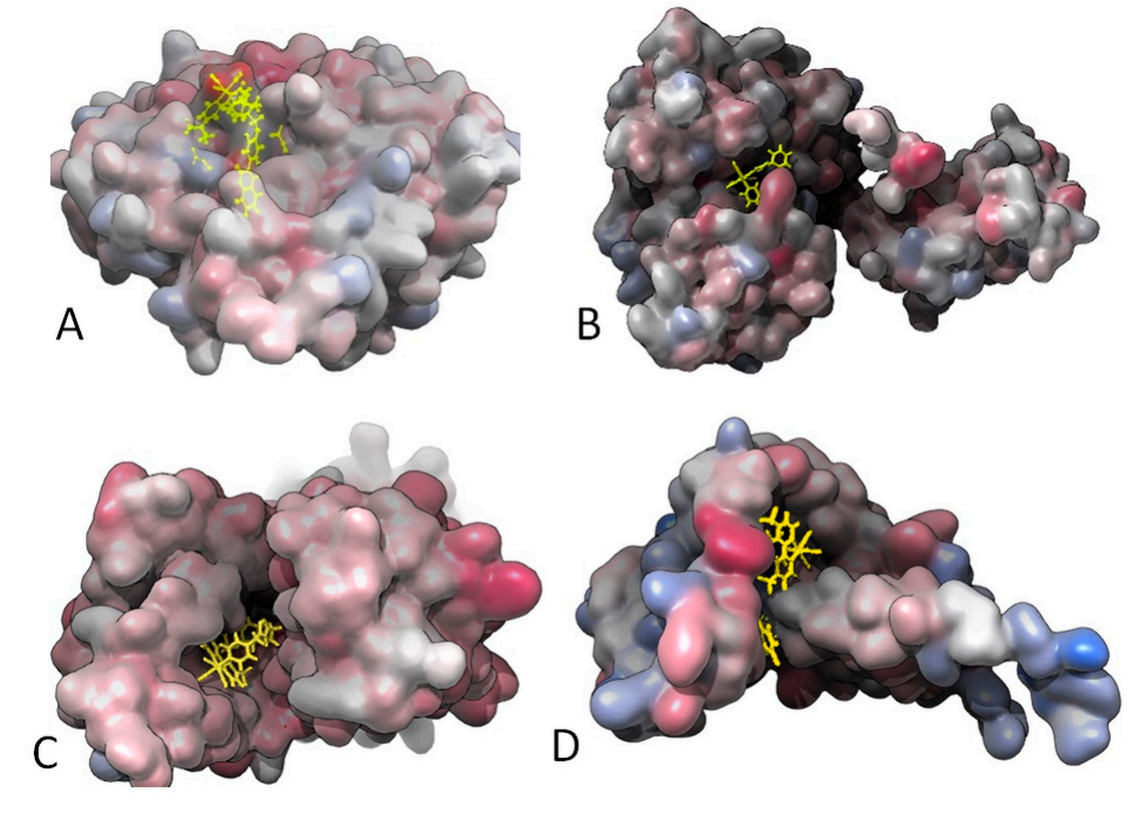
Gaussian surface representation of the hydrophobicity of (**A**) **18** and Lipoteichoic acid synthase (LtaS; 2W5Q); (**B**) **15** and type-I signal peptidase (SpsB; 4WVJ); (**C**) **19** and Lipoteichoic acid flippase (LtaA), top view (6S7V); and (**D**) **19** and Lipoprotein signal peptidase II (LspA; 6RYP). The red-blue color palette changes from hydrophilic blue to hydrophobic red.

Finally, in Figure 7 and Figure 8, the hydrophobic gaussian surface was used for the graphical representation of the binding pockets of the ligands. The hydrophobicity scales of Wimley and White were used for defining the hydrophobicity of the amino acid residues [90]. This prediction assumes that apolar sites are preferentially disposed to the molecular interior, forming a hydrophobic core, whereas polar sites are disposed outside the molecular interior. In the Supplementary Materials (Figure S8), the representation of the protein surface of the non-polar polar ratio (NPP) [91] and the patch analysis of the electrostatic surface potential are depicted [92]. To analyze the effect on the distortion of the receptor and the conformation changes when binding the complex, the results of the Rg for the receptors and the complexes are presented in Table 2. As can be understood from the values, the Rgs of the explored systems did not change significantly for any of the shown complexes. The solvent accessible surface area (SASA) was also assessed for all cases. We did not observe intrinsic flexibility changes of the receptor SASA and system SASA, which can also be seen from the data in the Table 2. We found, in most cases, that the interfaces gain accessibility in order to promote stable interactions. The localization of the complexes preserves the SASA, which is an indication of the protein stability in the presence and absence of the complexes (i.e., ligands). With this property, we have a clearer picture of the existing changes in the protein conformation. The available surface area is maintained before and after the docking, and, as intuitively predicted, the rhenium complexes prefer localizing in the hydrophobic pockets of the possible target enzymes (Figure 7 and Figure 8).

## 3. Conclusions

In this study, we reported the synthesis, characterization, and antimicrobial effects of a series of rhenium di- and tricarbonyl diimine complexes. Due to the lack of activity of the tested species, in an effort to identify the possible targets of the active complexes (and thus possibly understand the underlying reasons for the observed differences in the antimicrobial efficacy of Re complexes), we computationally evaluated the binding affinity of the active and inactive molecules against structurally characterized membrane-bound *S. aureus* proteins. Whereas the inactive compounds did not show an affinity for the enzymes, our docking protocol identified two possible major targets for some molecules of this class of compounds, namely lipoteichoic acid flippase (LtaA) and lipoprotein signal peptidase II (LspA). To our knowledge, our study is the first reported attempt to computationally identify MRSA biological targets of antibiotic rhenium complexes [93,94,95,96,97]. Experimental data are required in the future to confirm the in silico results, but our data are in line with the limited mechanistic studies that have previously been published on microbicidal rhenium species. Indeed, if the complexes inhibit the catalytic activity of LtaA and LspA, essential cell wall polymers cannot be assembled, leading to microbial death. We emphasize that LtaA and LspA may be targets for a fraction of known active antimicrobial Re complexes (namely **14**, **15**, **18**, and **19** in this study). The penicillin-binding protein 2a (PBP2a) might also be targeted by **15** and **19**, while MurG may be inhibited by **17**. We were not able to identify possible targets for compounds **14** and **16**; thus, their mechanism of action and targets remain unknown. We also showed that active rhenium complexes tend to localize in the hydrophobic pockets of target enzymes. In terms of the key molecular features common to active rhenium carbonyl complexes, our data support the notion that active diimine species are only cationic complexes of the *fac*-[Re(CO)_3_]^+^ core. If a CO ligand is substituted, leading to dicarbonyl *cis*-[Re(CO)_2_]^n^, regardless of the overall charge of the compounds, the molecules are devoid of any antimicrobial activity. Arguably, the most significant outcome of our study, i.e., the identification of LtaA and LspA as possible targets for this class of antibiotics, is that it offers the scientific community involved in this research support for the rational design of rhenium complexes based on the computational protocol for computer-aided drug design.

## 4. Materials and Methods

### 4.1. Reagents and Chemicals

All reagents and solvents were purchased from standard sources and used without further purification. The compound [Re(CO)_5_Br] was purchased from Sigma-Aldrich. Complexes (Et_4_N)[Re(CO)_2_Br_4_] [62], **1a**–**2a** [60], **1b**–**5b** [61], **1c** [98], **9a** [99], **9b** [100], **9c** [101], and **10** [49] were synthesized according to published procedures. Unless otherwise noted, the solvents used in the preparation of all molecules were dry and O_2_-free.

### 4.2. Instruments and Analysis

A Bruker Advance III 400 MHz spectrometer was used to record the NMR spectra. The ^1^H chemical shifts of molecules are reported relative to the residual solvent protons. A Bruker FTMS 4.7-T Apex II spectrometer was used to perform the mass analysis in the positive mode. UV-vis spectra were measured on a Thermo Fisher Scientific Jasco V730 spectrophotometer. A Bruker TENSOR II spectrometer was used to record the IR spectra, with the following parameters: 16 scans for the background, and 32 scans for a sample, with a resolution of 4 cm^−1^ in the 4000–600 cm^−1^ region. Single crystals were measured on a Stoe IPDS2 diffractometer (CuKα1 (λ = 1.5406 Å)) equipped with a cryostat from Oxford Cryosystems. The ShelXT structure solution program was used to solve the crystal structures. We used the Intrinsic Phasing refined with the ShelXL refinement package, using least squares minimization [102,103]. Data were deposited in the Cambridge Crystallographic Data Centre. The CCDC numbers are 2184717–2184724. The elemental analysis was performed using a LECO CHNS-932 elemental analyzer.

### 4.3. Synthetic Procedures

**(TDAE)[Re(CO)_2_(bpy)Br_2_]_2_ (1′)** was synthesized according to a similar published procedure [61]. Briefly, [Re(CO)_2_(bpy)Br_2_] (**1a**, 63.5 mg, 114.0 µmol) was dissolved in dry CH_2_Cl_2_ (DCM) (20 mL) in a glove box. Tetrakis(dimethylamino)ethylene (TDAE, 13.24 µL, 57.0 µmol, 0.5 eq.) was dissolved in dry DCM (1 mL). The latter solution was added dropwise to the solution of **1a**. The mixture was stirred under inert conditions for 15 min. The solvent was removed under reduced pressure, giving compound **1′** as a purple solid. Yield: 71.9 mg, 55.0 µmol, 96%. IR (cm^−1^), ν_CO_: 1861, 1775. UV-vis (DMF), λ_max_ [nm]: 593, 426, 308, 300. The results of the elemental analysis are as follows: calculated for C_34_H_40_Br_4_N_8_O_4_Re_2_: C, 31.01%; H, 3.06%; N, 8.51%; found: C, 30.71%; H, 2.94%; N, 7.89%.

**[Re(CO)_2_(bpy)(MeIm)Br] (3a)**. Degassed complex **1′** (35.6 mg, 27.0 µmol) was dissolved in dry toluene (20 mL). Anhydrous *N*-methyl imidazole (MeIm, 4.32 µL, 54 µmol, 2 eq.) was added, and the mixture was stirred at 100 °C for 48 h. The mixture was cooled to room temperature and the brown precipitate was isolated by centrifugation, giving compound **3a**. Yield: 15.8 mg, 28.1 µmol, 52%. Single crystals suitable for X-ray diffraction were grown by layering pentane on a CH_2_Cl_2_ solution of the compound, giving dark brown crystals. IR (cm^−1^), ν_CO_: 1877, 1779. UV-vis (DMSO), λ_max_ [nm]: 528, 375, 310, 303. ^1^H NMR (400MHz, CD_2_Cl_2_, ppm) δ: 9.06 (dd, J = 5.5, 1.1 Hz, 2 H), 8.70 (d, J = 8.2 Hz, 2 H), 8.30 (td, J = 7.9, 1.5 Hz, 2 H), 7.69 (ddd, J = 7.6, 5.6, 0.9 Hz, 2 H), 7.28 (s, 1 H), 6.79 (t, J = 1.4 Hz, 1 H), 6.59 (t, J = 1.5 Hz, 1 H), 3.57 (s, 3 H). ESI-MS (MeOH): *m/z*, 582.9 [M + Na]^+^. The results of the elemental analysis are as follows: calculated for C_16_H_14_BrN_4_O_2_Re: C, 34.29%; H, 2.52%; N, 10.00%; found: C, 34.70%; H, 2.40%; N, 9.52%.

**[Re(CO)_2_(bpy)(py)_2_]PF_6_ (4a)** was synthesized according to a similar published procedure [61]. Briefly, complex **2a** (54 mg, 97 µmol) and pyridine (py, 1 mL, ca. 100 eq.) were dissolved in MeOH (20 mL), and the mixture was stirred at 70 °C overnight. The solvent was removed under reduced pressure. The residue was dissolved in water (75 mL), and a solution of KPF_6_ (36 mg, 194 µmol, 2 eq.) in water (5 mL) was added dropwise to the rhenium. The precipitate was isolated by centrifugation, giving compound **4a** as a brown-orange solid. Yield: 45 mg, 64.1 µmol, 66%. Single crystals suitable for X-ray diffraction were grown by the diffusion of pentane into an acetone solution of the compound, giving dark orange crystals. IR (cm^−1^), ν_CO_: 1901, 1823. UV-vis (DMF), λ_max_ [nm]: 481, 357, 302. ^1^H NMR (400 MHz, CD_2_Cl_2_, ppm) δ: 9.32 (ddd, J = 0.73, 1.56, 5.41 Hz, 2H), 8.36–8.40 (m, 4H), 8.34 (d, J = 8.19 Hz, 2H), 8.18 (dt, J = 1.59, 7.95 Hz, 2H), 7.74 (ddd, J = 1.28, 5.47, 7.67 Hz, 2H), 7.59–7.65 (m, 2H), 7.05–7.11 (m, 4H). ^13^C NMR (101 MHz, CD_2_Cl_2_, ppm) δ: 205.9 (2C), 156.6 (2C), 155.3 (4C), 152.5 (2C), 141.1 (2C), 137.6 (2C), 129.3 (2C), 126.5 (4C), 125.1 (2C). ESI-MS (MeOH): *m/z*, 556.7 [M]^+^. The results of the elemental analysis are as follows: calculated for C_22_H_18_F_6_N_4_O_2_PRe: C, 37.66%; H, 2.59%; N, 7.99%; found: C, 37.53%; H, 2.72%; N, 7.67%.

**[Re(CO)_2_(bpy)(MeIm)_2_]PF_6_ (5a).** Compound **1′** (132 mg, 100 µmol) was dissolved in anhydrous MeIm (8 mL) and the mixture was stirred at 110 °C for 60 min. The solvent was removed under reduced pressure and the residue was purified by flash column chromatography (eluent: EtOAc 100%, then DCM/MeOH 100:0, increased to 98:2). The first fraction, compound **3a**, was collected with the first gradient (100% EtOAc) as a brown solid (amount: traces). The second fraction was collected with the last gradient as mobile phase. Once dried, the counter ion was exchanged with KPF_6_ (17.2 mg, 93.4 µmol) in H_2_O (15 mL). Complex **5a** was isolated by centrifugation as a violet solid. Yield: 24 mg, 33.9 µmol, 17%. IR (cm^−1^), ν_CO_: 1885, 1802. UV-vis (DMF), λ_max_ [nm]: 500, 363, 307, 300. ^1^H NMR (400 MHz, CD_2_Cl_2_, ppm) δ: 9.21–9.26 (m, 2H), 8.32 (dd, J = 0.86, 8.19 Hz, 2H), 8.12 (dt, J = 1.59, 7.95 Hz, 2H), 7.62 (ddd, J = 1.22, 5.44, 7.64 Hz, 2H), 7.29 (s, 2H), 6.63–6.67 (t, 2H), 6.44–6.51 (t, 2H), 3.53 (s, 6H). ^13^C NMR (101 MHz, CD_2_Cl_2_, ppm) δ: 207.6 (2C), 156.7 (2C), 152.5 (2C), 141.2 (2C), 140.4 (2C), 132.0 (2C), 128.6 (2C), 124.5 (2C), 122.3 (2C), 34.7 (2C). ESI-MS (MeOH): *m/z*, 562.7 [M]^+^. The results of the elemental analysis are as follows: calculated for C_20_H_20_F_6_N_6_O_2_PRe: C, 33.95%; H, 2.85%; N, 11.88%; found: C, 34.29%; H, 2.74%; N, 11.52%.

**(TDAE)[Re(CO)_2_(phen)Br_2_]_2_ (1″)** was synthesized according to a similar published procedure [61]. Briefly, cis-[Re(CO)_2_(phen)Br_2_] (62.8 mg, 107.9 µmol) was dissolved in dry DCM (17 mL) in a glove box. TDAE (12.56 µL, 53.9 µmol, 0.5 eq.) was dissolved in dry DCM (1.5 mL). The latter solution was added dropwise to the rhenium in the glove box, and the mixture was stirred for 15 min at room temperature. The solvent was removed under reduced pressure, giving **1″** as a brown-purple solid. Yield: 67.7 mg, 49.6 µmol, 92%. IR (cm^−1^), ν_CO_: 1856, 1771. 

**[Re(CO)_2_(phen)(py)Br] (2c)**. Degassed complex **1″** (20 mg, 14.7 µmol) was dissolved in degassed pyridine (2 mL), and the mixture was stirred at 100 °C for 20 min. The reaction mixture was cooled down to room temperature and extracted in DCM (50 mL) with HCL 0.1 M (3 × 50 mL). The organic phase was dried over sodium sulfate, and the solvent was removed under reduced pressure. The crude was purified by flash column chromatography (stationary phase: aluminum oxide, mobile phase: pentane/EtOAc/MeOH 1:2:0, increased to 0:1:0 and finally 0:99:1), giving compound **2c** as a brown solid. Yield: 0.9 mg, 1.6 µmol, 5%. IR (cm^−1^), ν_CO_: 1864, 1778. UV-vis (DMSO), λ_max_ [nm]: 511, 380, 269. ^1^H NMR (400MHz, CD_3_CN, ppm) δ: 9.61 (dd, J = 5.1, 1.2 Hz, 2H), 8.84 (dd, J = 8.3, 1.3 Hz, 2H), 8.25–8.29 (m, 2H), 8.17 (s, 2H), 8.11 (dd, J = 8.3, 5.1 Hz, 2H), 7.71–7.78 (m, 1H), 7.19 (s, 2H). The results of the elemental analysis are as follows: calculated for C_19_H_13_BrN_3_O_2_Re: C, 39.25%; H, 2.25%; N, 7.23%; found: C, 39.42%; H, 2.15%; N, 6.99%.

**[Re(CO)_2_(phen)(MeIm)Br] (3c)**. Degassed complex **1″** (17 mg, 12.5 µmol) was dissolved in anhydrous MeIm (2 mL) and the mixture was stirred at 110 °C for 20 min. The reaction mixture was cooled down to room temperature and extracted in DCM (50 mL) with HCl 0.1 M (3 × 50 mL). The organic phase was dried over sodium sulfate, and the solvent was removed under reduced pressure. The crude was purified by flash column chromatography (stationary phase: aluminum oxide, mobile phase: pentane/EtOAc 1:1, increased to 0:1), giving compound **3c** as a violet solid. Yield: 1.8 mg, 3.1 µmol, 12%. IR (cm^−1^), ν_CO_: 1876, 1773. UV-vis (DMSO), λ_max_ [nm]: 528, 275. ^1^H NMR (400MHz, (CD_3_)_2_SO, ppm) δ: 9.46–9.50 (m, 2H), 8.73–8.78 (m, 2H), 8.21 (s, 2H), 8.01–8.07 (m, 2H), 7.64–7.67 (m, 1H), 6.81–6.84 (m, 1H), 6.46–6.49 (m, 1H), 3.41 (s, 3H). The results of the elemental analysis are as follows: calculated for C_18_H_14_BrN_4_O_2_Re: C, 36.99%; H, 2.41%; N, 9.59%; found: C, 37.38%; H, 2.73%; N, 10.05%.

The following general procedure was applied for the synthesis of complexes **6**–**8** [50]. To a solution of [Re(CO)_5_Br] (1.0 equiv.) in hot toluene, the appropriate bipyridine (bpy) ligand (1.0 equiv.) was added, and the mixture was refluxed for 7–9 h. After the solution had cooled to the room temperature, the reaction mixture was filtered and washed with cold toluene (2×), yielding *fac*-[Re(CO)_3_(bpy)Br] as a bright fluorescent yellow powder. The solid was then dried in vacuo for 24h. The complexes were found to be pure (≥ 96%) by NMR and HPLC.

***fac*-[Re(CO)_3_(^t^Bu-bpy)Br] (6)**, where ^t^Bu-bpy is 4,4′-di-tert-butyl-2,2′-bipyridine. Pale yellow solid, yield 92%. IR (solid, cm^−1^); νCO: 2016, 1912, 1889, 1869. Uv-vis (DMF), λ_max_ [nm]: 368, 292. ^1^H-NMR (400 MHz, CD_3_CN, ppm): 8.96 (d, J = 5.99 Hz, 2H) 8.10 (d, J = 1.71 Hz, 2H) 7.51 (dd, J = 5.87, 1.96 Hz, 2H) 1.45 (s, 18H). ESI^+^-MS (MeOH): *m/z*, 576.9 [Re(CO)_3_(C_18_H_24_N_2_)(H_2_O)]^+^, [M-Br + H_2_O]^+^. Single crystals suitable for X-ray diffraction were grown by the diffusion of pentane into a DCM solution of the compound, giving yellow needles. The results of the elemental analysis are as follows: calculated for C_21_H_24_BrN_2_O_3_Re: C, 40.78%; H, 3.91%; N, 4.53%; found: C, 40.43%; H, 4.08%; N, 4.44%.

***fac*-[Re(CO)_3_(CF_3_-bpy)Br] (7)**, where CF_3_-bpy is 4,4′-bis(trifluoromethyl)-2,2′-bipyridine. Orange solid, yield 87%. IR (solid, cm^−1^); νCO: 2015, 1932, 1897. UV-vis (DMF), λ_max_ [nm]: 417, 304. ^1^H-NMR (400 MHz, CD_3_CN, ppm): 9.33 (d, J = 5.75 Hz, 2H) 8.46 (s, 2H) 7.84 (dd, J = 5.75, 1.22 Hz, 2H). ESI^+^-MS (MeOH): m/z, 580.7 [Re(CO)_3_(C_12_H_6_F_6_N_2_)(H_2_O)]^+^, [M-Br + H_2_O]^+^. Single crystals suitable for X-ray diffraction were grown by the diffusion of hexane into a DCM solution of the compound, giving orange needles. The results of the elemental analysis are as follows: calculated for C_15_H_6_BrF_6_N_2_O_3_Re: C, 28.05%; H, 0.94%; N, 4.36 %; found: C, 27.59%; H, 1.12%; N, 4.23%.

***fac*-[Re(CO)_3_((Et)_2_N-bpy)Br] (8)**, where (Et)_2_N-bpy is N4,N4,N4′,N4′-tetraethyl-[2,2′-bipyridine]-4,4′-diimine. Pale yellow solid, yield 92%. IR (solid, cm^−1^); νCO: 2008, 1886, 1866. UV-vis (DMF), λ_max_ [nm]: 367, 373. ^1^H-NMR (400 MHz, CD_3_CN, ppm): 8.49 (d, J = 6.60 Hz, 2H) 7.04 (d, J = 2.81 Hz, 2H) 6.54 (dd, J = 6.72, 2.69 Hz,2H) 3.49 (q, J = 7.21 Hz, 8H) 1.28 (t, J = 7.21 Hz, 12 H). ESI^+^-MS (MeOH): m/z, 568.9 [Re(CO)_3_(C_18_H_26_N_4_)]^+^, [M-Br]^+^. Single crystals suitable for X-ray diffraction were grown by the diffusion of pentane into a DCM solution of the compound, giving yellow needles. The results of the elemental analysis are as follows: calculated for C_21_H_26_BrN_4_O_3_Re: C, 38.89%; H, 4.04%; N, 8.64%; found: C, 38.45%; H, 4.17%; N, 8.47%.

***cis*-[Re(CO)_2_(batho-phen)Br_2_] (11).** Degassed (Et_4_N)[Re(CO)_2_Br_4_] (500 mg, 722 µmol) and batho-phen (240 mg, 722 µmol) were dissolved in dry DCM (80 mL). The mixture was stirred under inert conditions at room temperature for 72 h. The solvent was removed under reduced pressure, and the crude was purified by flash column chromatography on silica (Eluent: DCM / Pentane 1:9), giving complex **11** as an orange-red solid. Yield: 82 mg, 112 µmol, 15%. Single crystals suitable for X-ray diffraction were grown by the slow evaporation of DCM solution of the compound, giving dark brown needles. IR (cm^−1^), ν_CO_: 1999, 1849. UV-Vis (DMF), λ_max_ [nm]: 429, 288. The results of the elemental analysis are as follows: calculated for C_26_H_16_Br_2_N_2_O_2_Re: C, 42.52%; H, 2.20%; N, 3.81%; found: C, 42.00%; H, 2.42%; N, 3.84%.

### 4.4. Biological Tests

The culture conditions and in vitro antimicrobial activity determination were performed exactly as previously reported [60]. We thank Dr Aleksandra Barac (University Clinical Center of Serbia) and Prof. Cornelia Lass-Floerl (University of Innsbruck) for kindly providing the clinical *C. auris* strain 7. All other microbial strains (*Enterobacter cloacae* ATCC 3047, *Klebsiella pneumoniae* ATCC 13803, *Acinetobacter baumannii* ATCC 19606, *Pseudomonas aeruginosa* PAO1 NCTC10332, *Staphylococcus aureus* MRSA43300 (methicillin-resistant) and *S. aureus* ATCC25923 (methicillin-sensitive), and *Candida albicans* SC5314) were obtained from the American Type Culture Collection (ATCC) and the National Collection of Type Cultures (NCTC).

### 4.5. In Silico Calculations

#### 4.5.1. Preparation of the Ligand Database and Ligands: Receptors Complexes

The docking calculations were performed with AutoDock Vina version 1.2.0 (The Scripps Research Institute, La Jolla, San Diego, CA, USA) [14] and AutoDock4 version 4.2.6 (AD4, The Scripps Research Institute, La Jolla, San Diego, CA, USA) [104]. The receptor/protein PDBQT files were prepared, and the grid box size was determined using the AutoDock Tools version 1.5.7 (ADT, Scripps Research Institute, La Jolla, San Diego, CA, USA) [104]. Biovia Discovery Studio Visualizer 2021, version 21.1.0.20298 (Dassault Systèmes, San Diego, California, CA, USA), was used to visualize the receptor and ligand interactions. Figures were prepared with the ADT software. The structures of complexes **5–11** and **15** were obtained by the determined X-ray structures. Chemical structures as .CIF files were converted to .MOL2 files using the Mercury (Build RC1) software, version 3.7 (CCDC 2001–2015). All complexes (ligands) were optimized with the hybrid meta-GGA functional wB97XD [105,106,107,108,109], designed to account for dispersion, which was used in combination with the standard SDD basis sets [110]. The optimized structures were subject to frequency analysis to verify that they represented minima on the potential energy surface. All calculations were performed with Gaussian 09 software (version 5.0.9, Carnegie Mellon University, Gaussian, Inc., Wallingford, CT, USA).

The ADT software was then used to investigate the complexes’ structures in terms of their combinations with nonpolar hydrogens, additions of Gasteiger changes, and rotatable bonds. The rhenium atom is not parametrized in AD4 and AutoDock Vina; thus, AutoDock Vina calculations were performed using Mn instead of Re. The resulting binding poses of the Mn complexes were then cross-checked with the corresponding Re complexes using AD4, where the following line was added to the AD4 atom parameters file: “atom_par Re 2.95 0.066 12.000 -0.00110 0.0 0.0 0 -1 -1 1 # Non-H-bonding”. The binding poses of the Mn and Re complexes were found to be the same. Moreover, due to the fact that ADT failed to assign a Gasteiger change to the metal ion, a charge of 0.320 (to either Mn or Re) was assigned to the atom by editing the corresponding .PDBQT file [111].

The crystal structure of *S. aureus* proteins were obtained from the RCSB protein data bank (http://www.rcsb.org). Only the structures of membrane protein annotation (PDBTM, MemProtMD, OPM or mpstruc) were considered and selected. All water molecules were removed, and the required files for AutoDock Vina and AD4 were prepared by assigning hydrogens and Kollman charges to the protein structures, and finally converting them from the .PDB file format to .PDBQT file format.

#### 4.5.2. Molecular Docking

The docking calculations were conducted using the AutoDock Vina software (https://vina.scripps.edu/) with adapted parameters for the rhenium complexes. The extended version of the Vina code was used via the integrated platform SAMSON [https://www.samson-connect.net] as a SAMSON extension [112]. This provides additional functionality when preparing receptors and ligands, docking libraries, and analyzing and exporting docking results. Both the number of flexible side chains and the size of the search domain were different for all the cases because of the receptor’s conformation (i.e., the chain orientation and position of residues). On average, there were about 30 flexible side chains with unlocked rotatable bonds. The search space was defined by a docking box wrapper of the space around the receptors. The scaling of the box depended on the defined pocket score. The number of modes was set to 200 with an energy range = 3 kcal/mol (default value). The energy range is the maximum energy difference between the best binding mode and the unfavorable one displayed (kcal/mol). Energy (affinity) values that differed by more than 3 kcal/mol from the best mode were not saved among the results. In the configuration file, the parameter called “exhaustiveness” was set to 40. This parameter controls how comprehensive the search space is. In AutoDock Vina, the electrostatic interactions were handled with the hydrophobic and the hydrogen bonding terms. The post-docking analysis of the favorable ligand–receptor complexes was performed via the Protein–Ligand Interaction Analyzer Extension in SAMSON [112]. With the help of the Protein–Ligand Interaction Analyzer, it was possible to calculate the radius of gyration, hydrogen bonds, residues surrounding the ligand, and solvent-accessible surface area (SASA) of the receptor and ligand and of the form complexes. The multistep validation protocol was considered in this study, and the ability of the combined methodology was examined independently through initial screening and extensive semi-flexible docking.

## Figures and Tables

**Table 1:** Antimicrobial activity, assessed by determining the minimal inhibitory concentrations (MIC, µM, mean of n = 3 independent experiments) of different Re-bearing complexes.

Compound.	*A. baumannii*	*P. auruginosa*	*K. pneumoniae*	*S. aureus* MRSA	*S. aureus* MSSA	*E. cloacae*	*C. albicans*	*C. auris*
**1a–5a**	>100	>100	>100	>100	>100	>100	>100	>100
**1b–3b**	>100	>100	>100	>100	>100	>100	>100	>100
**4b**	>100	>100	>100	25	25	>100	>100	>100
**5b**	>100	>100	>100	50	50	>100	>100	>100
**1c–3c**	>100	>100	>100	>100	>100	>100	>100	>100
**6–8, 9a–c, 10**	>50	>50	>50	>50	>50	>50	>50	>50
**11**	>100	>100	>100	50	50	>100	>100	>100
**13**	n.a.	n.a.	n.a.	0.7	22.8	n.a.	n.a.	n.a.
**14**	n.a.	n.a.	n.a.	1.6	1.6	n.a.	n.a.	n.a.
**15**	n.a.	n.a.	n.a.	0.4	0.6	n.a.	6.2	50
**16**	n.a.	n.a.	n.a.	0.8	0.8	n.a.	6.2	50
**17**	8	32	32	0.25	0.25	n.a.	n.a.	n.a.
**18**	n.a.	n.a.	n.a.	0.8	3.1	n.a.	3.1	50
**19**	n.a.	n.a.	n.a.	1.6	6.2	n.a.	50	50
Meropenem	>100	1.25	>100	6.25	>100	50	-	-
Vankomycin	>100	>100	>100	6.25	6.25	-	-	-
Fluconazole	-	-	-	-	-	-	1	>64
Amphotericin B	-	-	-	-	-	-	0.3	1

Note: n.a. = not available.

**Table 2:** Drug-likeness properties of active antimicrobial rhenium complexes **13–19**.

Compound	MW	RBN	TPSA(Tot)	HBA	HBD	LOGP99	BLTF96	BLTA96	BLTD48	ESOL	cRo5	Ro5
**13**	700.827	3	81.79	6	1	6.8	−3.09	−3.22	−3.23	−7.19	1	0
**14**	1072.267	16	187.36	14	2	4.8	1.13	1.49	1.73	−5.67	0	1
**15**	681.767	3	66	0	6	6.7	−2.99	−3.11	−3.11	−7.02	1	0
**16**	647.827	7	72.48	0	8	4.1	−2.03	−2.04	−1.98	−4.94	1	0
**17**	771.297	5	70.93	8	0	7.1	−3.94	−4.17	−4.23	−7.85	1	0
**18**	867.067	3	108.24	2	1	8.5	−4.13	−4.38	−4.45	−8.74	1	0
**19**	1021.367	8	136.15	0	12	8.0	−3.84	−4.06	−4.11	−9.29	0	1

Labels: MW—molecular weight, RBN—rotatable bond number, TPSA—total polar surface area in A^2^, HBA—number of H bond acceptors, HBD—number of H bond donors, LOGP99—Wildman–Crippen octanol–water partition coeff., BLTF96—Verhaar Fish base-line toxicity from MLOGP (mmol/L), BLTD48—Verhaar Daphnia base-line toxicity from MLOGP (mmol/L), BLTA96—Verhaar Algae base-line toxicity from MLOGP (mmol/L), ESOL—estimated solubility (logS) for aqueous solubility using LOGPcons., cRo5—complementary Lipinski alert index, Ro5—Lipinski rule of 5.

**Table 3:** Predicted binding sites.

PBD ID	Area (Å^2^)	Volume (Å^3^)	Pocket Residues ID/Flexible Chains
**2OLV** (PBP2)	3500.5	7715.2	ALA_112, VAL_367, GLY_339, LYS_127, LYS_135, THR_150, VAL_153, THR_148, GLU_171, LYS_194, PRO_231, ASN_193, GLY_229
**4DKI** (PBP2a)	5537.8	9122.9	THR_398, PRO_401, VAL_443, THR_444, SER_461, TYR_519, GLY_520, THR_582, ALA_601, ARG_612, ASP_638
**3VSL** (PBP3)	9921.9	13845.0	GLY_424, VAL_390, LEU_425, THR_426, MET_453, LEU_518, ASP_519, LYS_618, TYR_636
**5TXI** (PBP4)	4258.4	4521.5	SER_75, ALA_74, THR_77, LYS_78, SER_137, SER_185, SER_262, PHE_241, THR_260, GLY_261, PRO_113, LEU_115, GLU_114
**2W5Q** (LtaS)	132.1	103.4	LEU_254, GLU_255, GLN_297, GLY_298, LYS_299, THR_300, SER_301, HIS_347, PHE_353, TRP_354, ASN_355, LYS_397, HIS_416
**4WVJ** (SpsB)	1922.1	3375.8	TRP_236, GLU_117, GLU_159, TYR_161, ASN_18, ASP_20, LYS_21, LEU_268, SER_343, TRP_346, TYR_347, ARG_350, LYS_48
**6S7V** (LtaA)	1758.2	2257.5	LEU_219, PRO_221, LEU_225, ALA_229, ILE_230, ALA_230, VAL_234
**6RYP** (LspA)	8452.7	1485.4	ALA_103, _367, GLY_339, LYS_127, LYS_135, THR_150, VAL_153, THR_148, GLU_171, LYS_194, PRO_231, ASN_193, GLY_229

**Table 4:** Molecular docking scores and related properties.

Receptor	Drug	Affinity *	H-Bonds	Receptor’s Rgyr (nm)	System’s Rgyr (nm)	Receptor’s SASA (nm^2^)	Receptor’s Prob. Drugability	Ligand’s SASA (nm^2^)	System’s SASA (nm^2^)	Contact Area (nm^2^)	Detected H-Bonds with AA Residue
2OLV (PBP2)	**13**	−6.9	3	3.29	3.29	295.26	0.82	7.60	295.24	3.80	ASP 156, LYS 194, PRO 231
	**14**	−7.4	3	3.29	3.29	295.26	0.82	12.14	294.91	6.24	ASP 156, LYS 194, PRO 231
	**15**	−8.1	2	3.29	3.28	295.26	0.82	7.87	294.87	4.13	ASP 89
	**16**	−5.7	4	3.29	3.28	295.26	0.82	8.05	295.15	4.08	THR 87, GLN 92, HIS 94, GLU 95
	**17**	−7.8	1	3.29	3.32	295.26	0.82	7.90	299.57	3.13	ASP 156
	**18**	−7.9	1	3.29	3.29	295.26	0.82	10.77	295.17	5.43	PRO 72
	**19**	−7.2	1	3.29	3.30	295.26	0.82	9.35	295.14	4.73	ASN 237
4DKI * (PBP2a)	**13**	−6.7	1	3.66	3.66	317.73	0.76	7.09	316.08	4.10	THR 398
	**14**	−7.2	3	3.66	3.65	317.73	0.76	11.21	316.08	6.43	THR 398, GLY 520
	**15**	−9.2	1	3.66	3.66	317.73	0.76	7.55	315.86	4.71	LYS 394
	**16**	−5.9	4	3.66	3.66	317.73	0.76	7.87	316.27	4.66	THR 600, LEU 603, MER 605
	**17**	−6.7	4	3.66	3.67	317.73	0.76	7.7	317.2	3.9	ASP 516, GLN 521, MET
	**18**	−8.5	1	3.66	3.66	317.73	0.76	10.75	316.08	6.94	SER 400
	**19**	−9.8	4	3.66	3.67	317.73	0.76	11.57	315.25	7.02	SER 403, GLN 521, THR 600, SER 400
3VSL (PBP3)	**13**	−7.0	3	3.11	3.11	301.97	0.81	7.25	300.48	4.37	TYR 525, GLU 623, GLN 626
	**14**	−7.0	3	3.11	3.11	301.97	0.81	12.00	299.12	7.42	
	**15**	−8.6	3	3.11	3.11	301.97	0.81	7.80	300.81	4.48	TYR 525, ASP 519, GLU 623
	**16**	−5.3	1	3.11	3.11	301.97	0.81	8.01	301.13	4.42	GLN 626
	**17**	−6.9	0	3.11	3.11	301.97	0.81	7.69	300.78	4.44	-
	**18**	−7.6	2	3.11	3.11	301.97	0.81	11.22	302.30	5.44	GLU 623
	**19**	−6.7	3	3.11	3.11	301.97	0.81	11.88	301.39	6.23	GLU 623
5TXI * (PBP4)	**13**	−6.3	0	2.16	2.17	151.84	0.8	7.7212	155.88	1.83	-
	**14**	−9.1	5	2.16	2.16	151.84	0.8	11.821	150.85	6.40	GLU 114, SER 262, TYR 268, TYR 291, GLU 297
	**15**	−7.0	0	2.16	2.17	151.84	0.8	7.951	156.74	1.5	-
	**16**	−5.6	0	2.16	2.16	151.84	0.8	7.9606	150.89	4.45	-
	**17**	−7.1	2	2.16	2.17	151.84	0.8	7.6	155.2	2.6	THR 240, GLY 247
	**18**	−8.1	3		2.16	151.84	0.8	10.5309	151.17	5.60	GLU 114, SER 262, TYR 268, TYR 291
	**19**	−10.02	3	2.16	2.16	151.84	0.8	12.6803	151.04	6.73	SER 116
2W5Q (LtaS)	**13**	−6	1	2.07	2.06	177.84	0.81	7.25	178.03	3.53	ASP 502
	**14**	−6.2	0	2.07	2.07	177.84	0.81	10.73	177.85	5.36	-
	**15**	−7.8	1	2.07	2.07	177.84	0.81	7.99	178.05	3.89	ASP 366
	**16**	−5.7	0	2.07	2.06	177.84	0.81	7.94	176.11	4.83	-
	**17**	−7.5	1	2.07	2.07	177.84	0.81	7.72	184	0.7	ASP 521
	**18**	−8.9	2	2.07	2.06	177.84	0.81	10.98	176.25	6.28	GLY 296, GLY 478
	**19**	−7.5	0	2.07	2.0697	177.84	0.81	9.2	177.15	4.98	-
4WV J* (SpsB)	**13**	−7.3	2	2.77	2.75	239.62	0.82	7.26	238.84	4.02	SER 343
	**14**	−8.3	2	2.77	2.75	239.62	0.82	12.90	237.89	7.31	TYR 182, ALA 330
	**15**	−9.5	2	2.77	2.76	239.62	0.82	7.76	238.10	4.64	ASP 20
	**16**	−6.1	0	2.77	2.75	239.62	0.82	8.09	237.67	5.02	-
	**17**	−7.1	0	2.77	2.76	239.62	0.82	7.70	238.36	4.1	-
	**18**	−7.5	2	2.77	2.75	239.62	0.82	8.89	238.49	5.01	GLU 51, PRO 340
	**19**	−8.9	2	2.77	2.74	239.62	0.82	10.14	238.36	5.70	GLU 50, VAL 378
6S7V * (LtaA)	**13**	−8.3	1	2.13	2.12	192.79	0.81	7.55	190.60	4.87	GLY 259
	**14**	−8.6	1	2.13	2.12	192.79	0.81	11.06	187.8256	8.01	ILE 256
	**15**	−10.0	1	2.13	2.12	192.79	0.81	7.91	190.39	5.15	TYR 377
	**16**	−6.2	0	2.13	2.12	192.79	0.81	7.84	190.33	5.15	-
	**17**	−8.0	0	2.13	2.12	192.79	0.81	7.8	189.5	3.8	-
	**18**	−9.7	0	2.13	2.12	192.79	0.81	11.06	189.55	7.15	-
	**19**	−10.2	2	2.13	2.12	192.79	0.81	9.71	189.48	6.51	ILE 230, TYR 377
6RYP * (LspA)	**13**	−7.4	1	1.86	1.84	108.41	0.82	7.31	107.33	4.19	GLY 54
	**14**	−10.0	2	1.86	1.83	108.41	0.82	12.55	105.94	7.51	ASP 136
	**15**	−10.6	0	1.86	1.84	108.41	0.82	7.91	106.88	4.71	-
	**16**	−7	0	1.86	1.84	108.41	0.82	7.82	105.99	5.15	-
	**17**	−8.1	2	1.86	1.85	108.41	0.82	7.5	107.3	4.05	ILE 120, THR 140
	**18**	−9.2	2	1.86	1.83	108.41	0.82	9.76	106.50	5.83	GLY 54, THR 140
	**19**	−11.5	1	1.86	1.83	108.41	0.82	10.58	106.47	6.26	THR 140

* Framed values indicate relative binding affinities < −9.0 kcal/mol.

## Data Availability

Not applicable.

## Supplementary Materials

The following supporting information can be downloaded at: https://www.mdpi.com/article/10.3390/ph15091107/s1, Figures S1–S8: 1H-NMR spectra of compounds; Figure S9: IR spectra (solid state) of compounds; Figure S10: UV-vis spectra (in DMF) of compounds; Figure S11: Visualization of the surface protein surface polarity: (A) non-polar to polar SASA color-coded from low NPP ratio (purple) to high NPP ratio (green); and (B) color-coded from negative charge (red) to positive charge (blue). Regions of high hydrophobicity are colored green, low hydrophobicity are colored purple; Figure S12a: Binding orientation of the compounds with hydrogen–acceptor and hydrogen–donor distances: A. 15 and PBP2: 2OLV; B. 19 and PBP2a: 4DKI; C. 15 and PBP3: 3VSL; D. 19 and PBP4: 5TXI; Figure S12b: Binding orientation of the compounds with hydrogen–acceptor and hydrogen–donor distances: A. 18 and lipoteichoic acid synthase (LtaS): 2W5Q; B. 15 and type-I signal peptidase (SpsB): 4WVJ; C. 19 and lipoteichoic acid flippase (LtaA): 6S7V; D. 19 and lipoprotein signal peptidase II (LspA): 6RYP; Table S1a: In silico pre-screening of binding affinities (b.a.; docking scores, kcal/mol) of rhenium complexes against structurally characterized membrane-bound S. aureus proteins: penicillin-binding proteins (PBPs); Table S1b: In silico pre-screening of binding affinities (b.a.; docking scores, kcal/mol) of non-toxic complexes against other structurally characterized membrane-bound S. aureus proteins; Table S2: Pockets prediction: mapping the ranking with residue environment distribution; Table S3: Percentage distribution of the surrounding residue types for the two groups of proteins; Table S4a–S4i: Selected bond lengths of the complexes; Table S4a’–S4i’: Selected bond angles of the complexes.
