# Supplementary material for: Antimicrobial Activity of Rhenium Di- and Tricarbonyl Diimine Complexes: Insights on Membrane-Bound S. aureus Protein Binding
Source: Pharmaceuticals (Basel). 2022 Sep 5;15(9):1107. doi: 10.3390/ph15091107 (PMC9501577; doi:10.3390/ph15091107)
Supplement: Supplementary file 1 [file pharmaceuticals-15-01107-s001.zip › pharmaceuticals-1876815-supplementary.pdf]

pharmaceuticals

Article

Supporting Information for:

Antimicrobial Activity of Rhenium Di- and Tricarbonyl Diimine Complexes: Insights on Membrane-Bound *S. aureus* Proteins Binding

Kevin Schindler [1,†], Youri Cortat [1,†], Miroslava Nedyalkova [1], Aurélien Crochet [1], Marco Lattuada [1], Aleksandar Pavic [2,*] and Fabio Zobi [1,*]

[1] Department of Chemistry, Fribourg University, Chemin Du Musée 9, 1700 Fribourg, Switzerland
[2] Institute of Molecular Genetics and Genetic Engineering, University of Belgrade, Vojvode Stepe 444a, 11042 Belgrade, Serbia
* Correspondence: sasapavic@imgge.bg.ac.rs (A.P.); fabio.zobi@unifr.ch (F.Z.)
† These authors contributed equally to this paper

Table of contents

1H-NMR spectra of compounds–**Figures S1–S8**
IR spectra (solid state) of compounds–**Figure S9**
UV-Vis spectra (in DMF) of compounds–**Figures S10**
In silico pre-screening of binding affinities (*b.a.*; docking scores. kcal/mol) of rhenium complexes against structurally characterized membrane bound *S. aureus* proteins: Penicillin Binding Proteins (PBPs).–**Table S1a**
In silico pre-screening of binding affinities (b.a.; docking scores. kcal/mol) of non-toxic complexes against other structurally characterized membrane bound *S. aureus* proteins.–**Table S1b**
Visualisation of surface protein surface polarity (A) non-polar to polar SASA colour-coded from low NPP ratio (purple) to high NPP ratio (green), and in (B) colour-coded from negative charge (red) to positive charge (blue). Regions of high hydrophobicity are coloured green, low hydrophobicity coloured purple.–**Figure S11**
Pockets prediction–mapping the ranking with residues environment distribution–**Table S2**
Binding orientation of the compounds with hydrogen-acceptor and hydrogen-donor distances: A. **15** and PBP2 : 2OLV ; B. **19** and PBP2a : 4DKI ; C. **15** and PBP3: 3VSL; D. **19** and PBP4: 5TXI.–**Figures S12a**
Binding orientation of the compounds with hydrogen-acceptor and hydrogen-donor distances: A. **18** and Lipoteichoic acids synthase (LtaS): 2W5Q; B. **15** and Type-I signal peptidase (SpsB): 4WVJ; C. **19** and Lipoteichoic acids flippase (LtaA): 6S7V; D. **19** and Lipoprotein signal peptidase II (LspA): 6RYP.–**Figures S12b**
Percentage distribution of the surrounding residue types for the two groups of protein.–**Table S3**
Selected Bond Lengths for complexes.–Table S4a-S4i
Selected Bond Angles for complexes.–Table S4a'-S4i'
References–page 24

NMR spectra

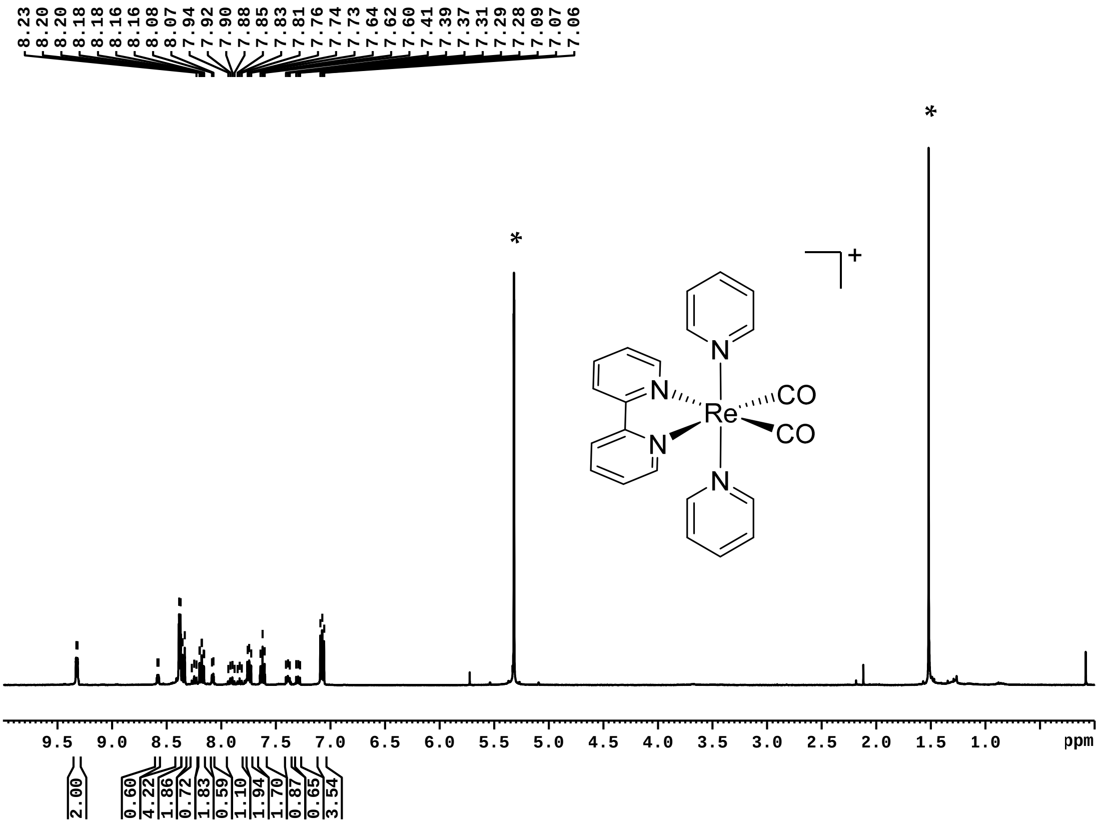

Figure S1. ^{1}H NMR spectrum of **4a** in CD_2Cl_2, ✱ = solvent residual peak (DCM, H_2O).

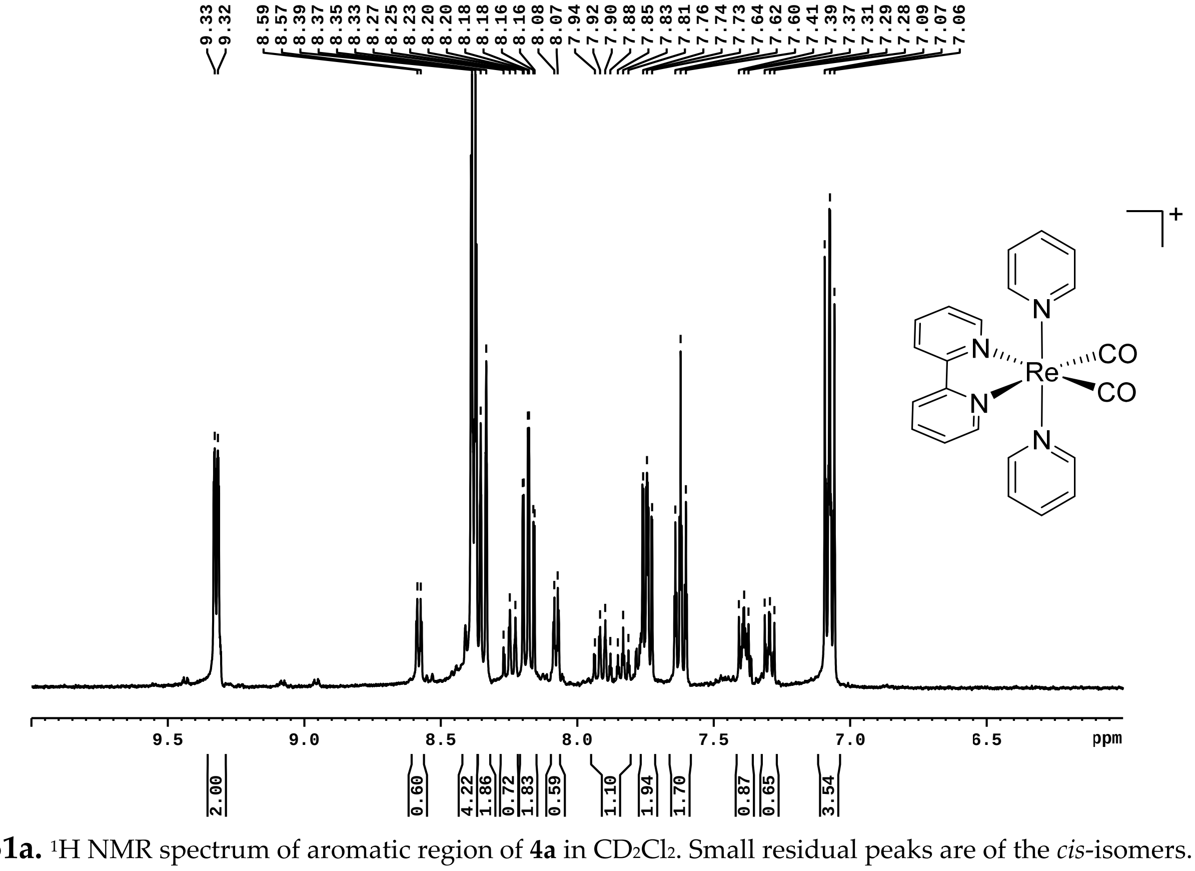

Figure S1a. ^{1}H NMR spectrum of aromatic region of **4a** in CD_2Cl_2. Small residual peaks are of the *cis*-isomers.

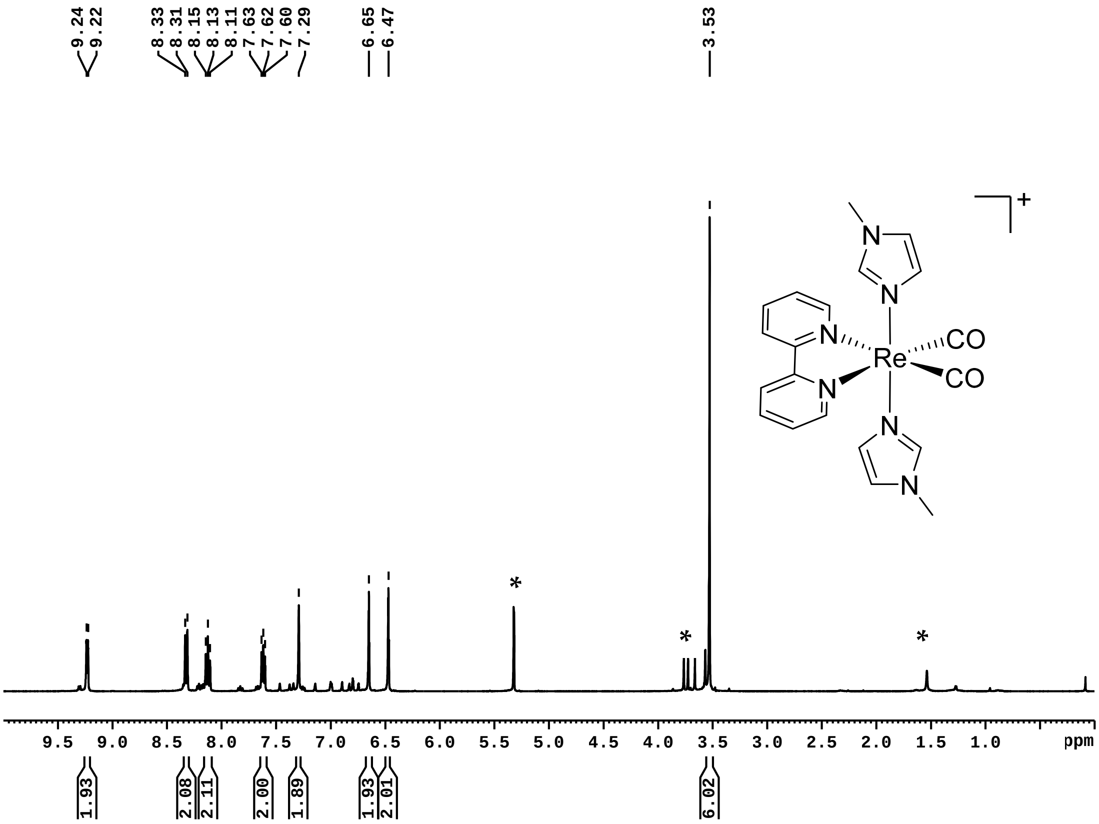

Figure S2. ^1H NMR spectrum of **5a** in CD_2Cl_2, ✱= solvent residual peak (DCM, H_2O, MeIm).

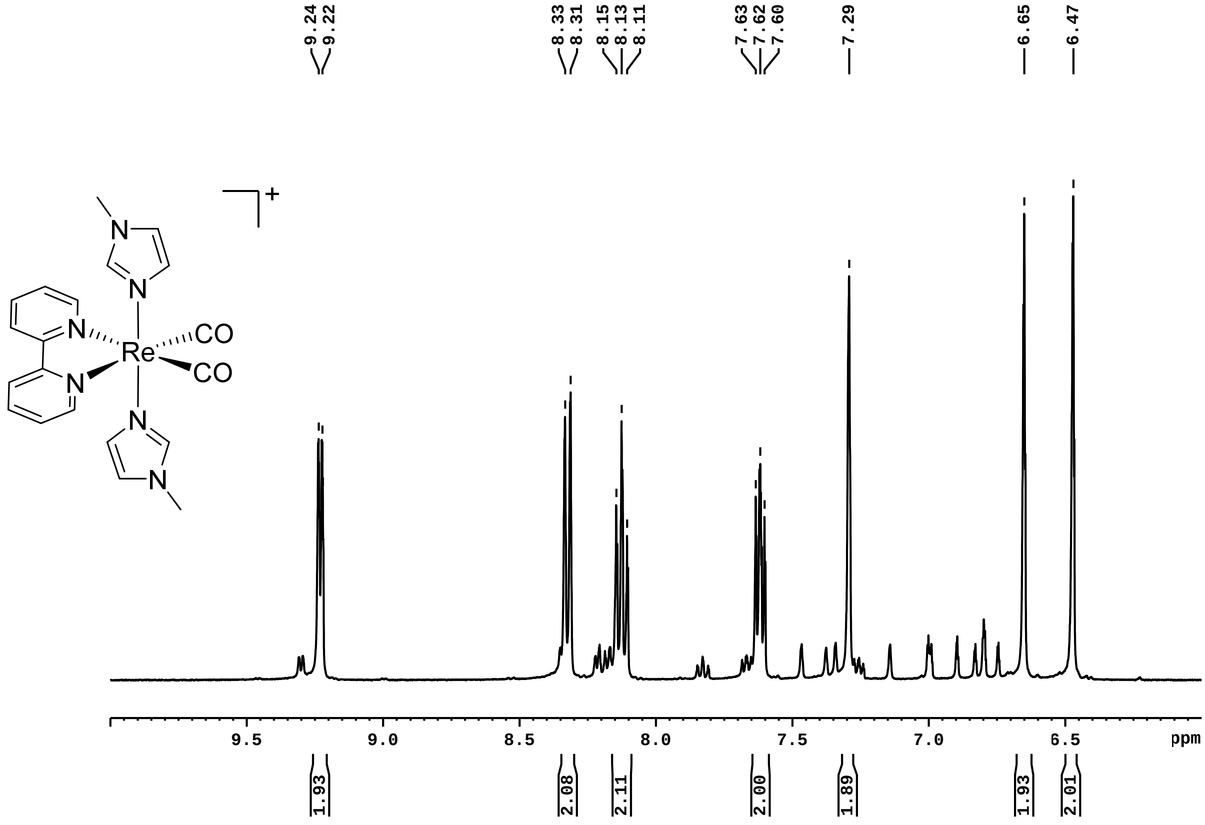

Figure S2a. ^1H NMR spectrum of aromatic region of **5a** in CD_2Cl_2. Small residual peaks are of the *cis*-isomers.

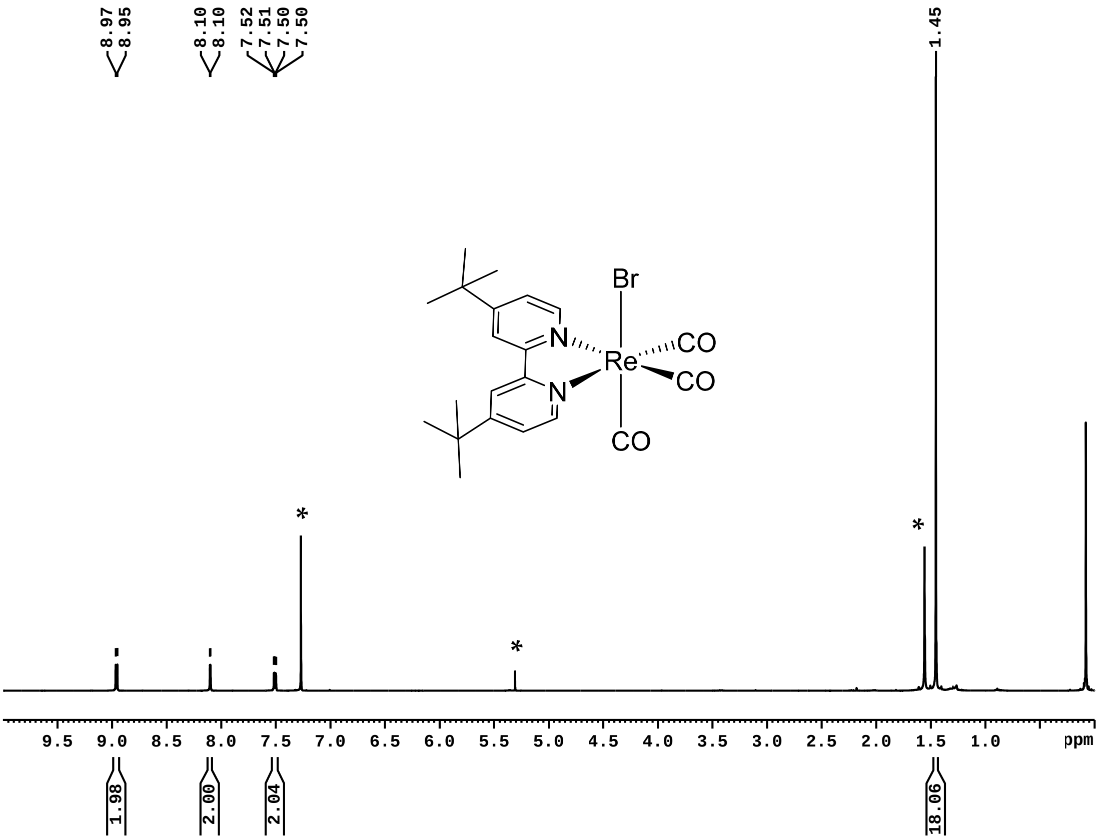

Figure S3. ¹H NMR spectrum of **6** in $CDCl_3$, ✱= solvent residual peak ($CHCl_3$, H_2O, DCM).

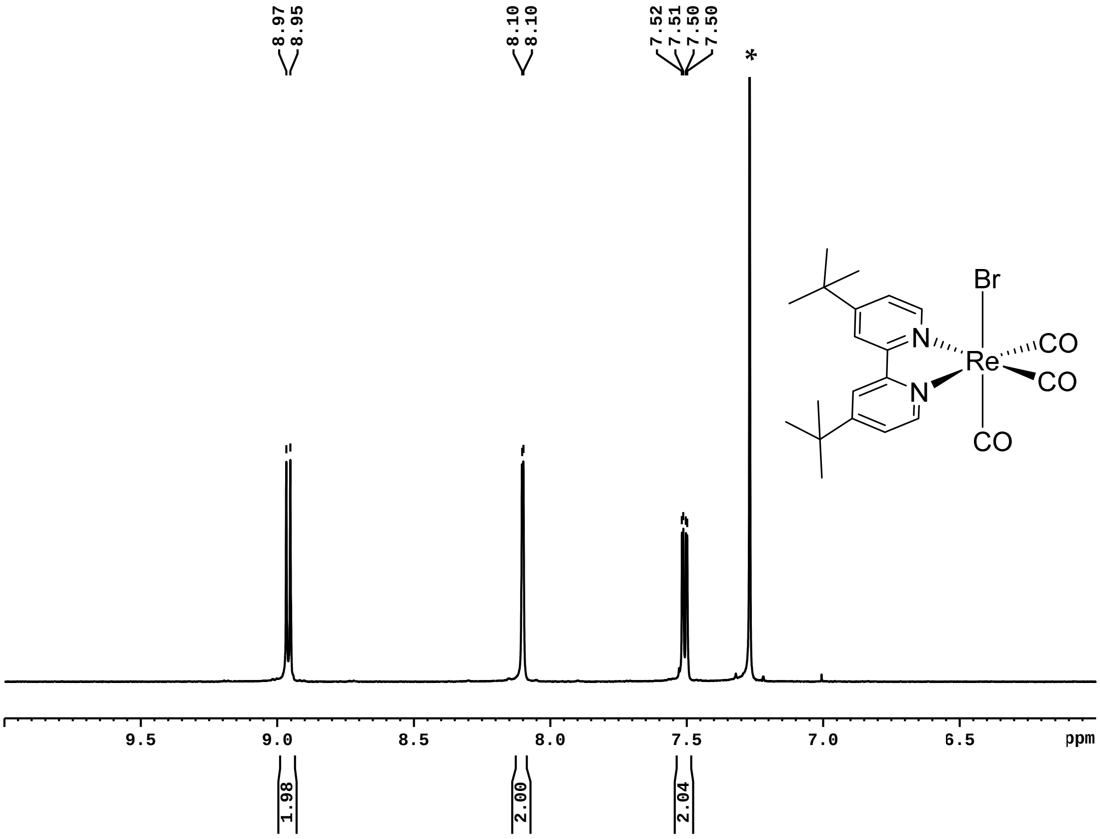

Figure S3a. ¹H NMR spectrum of aromatic region of **6** in $CDCl_3$ (zoom), ✱= solvent residual peak ($CHCl_3$).

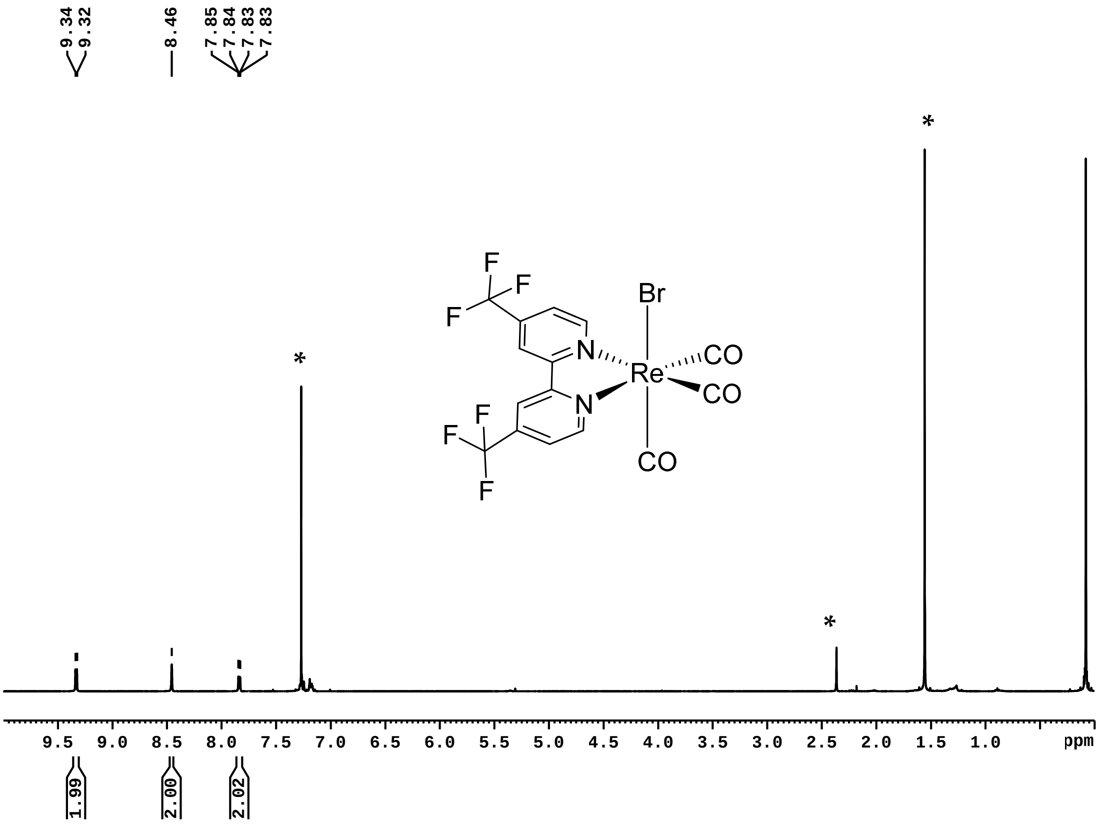

Figure S4. ^{1}H NMR spectrum of **7** in $CDCl_3$, ✱= solvent residual peak ($CHCl_3$, H_2O, Tol).

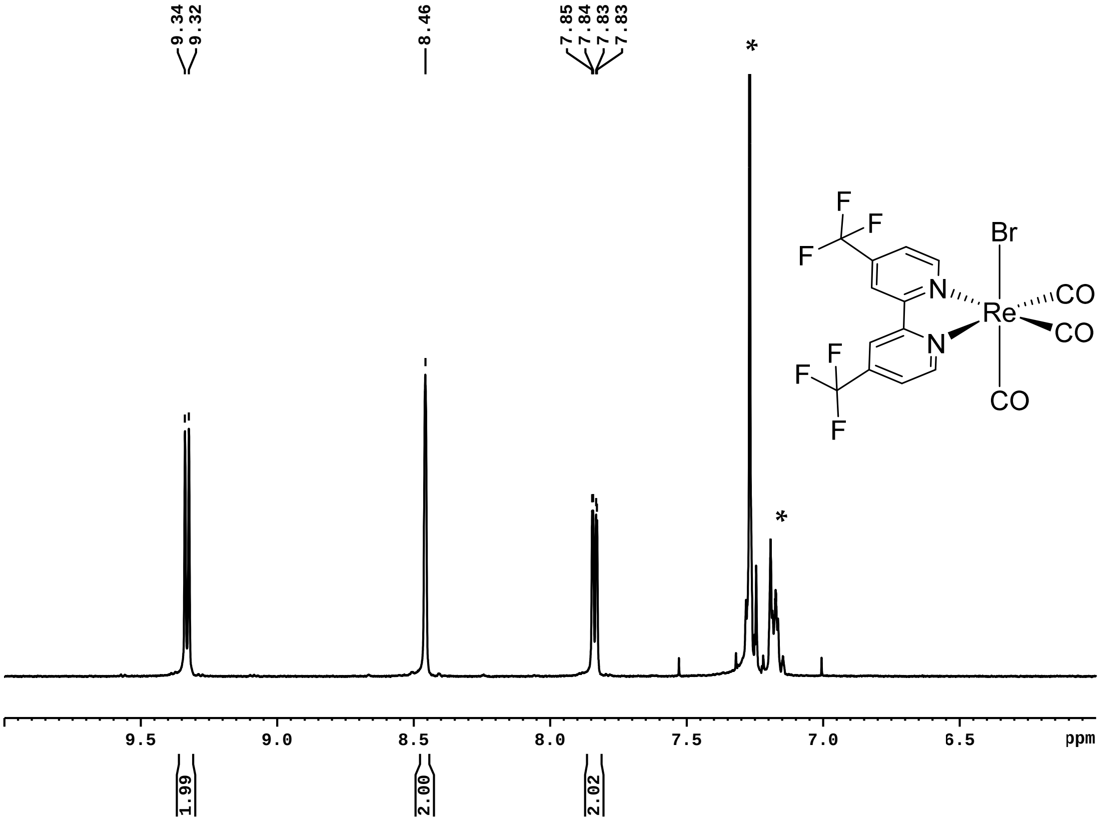

Figure S4a. ^{1}H NMR spectrum of aromatic region of **7** in $CDCl_3$ (zoom), ✱= solvent residual peak ($CHCl_3$, Tol).

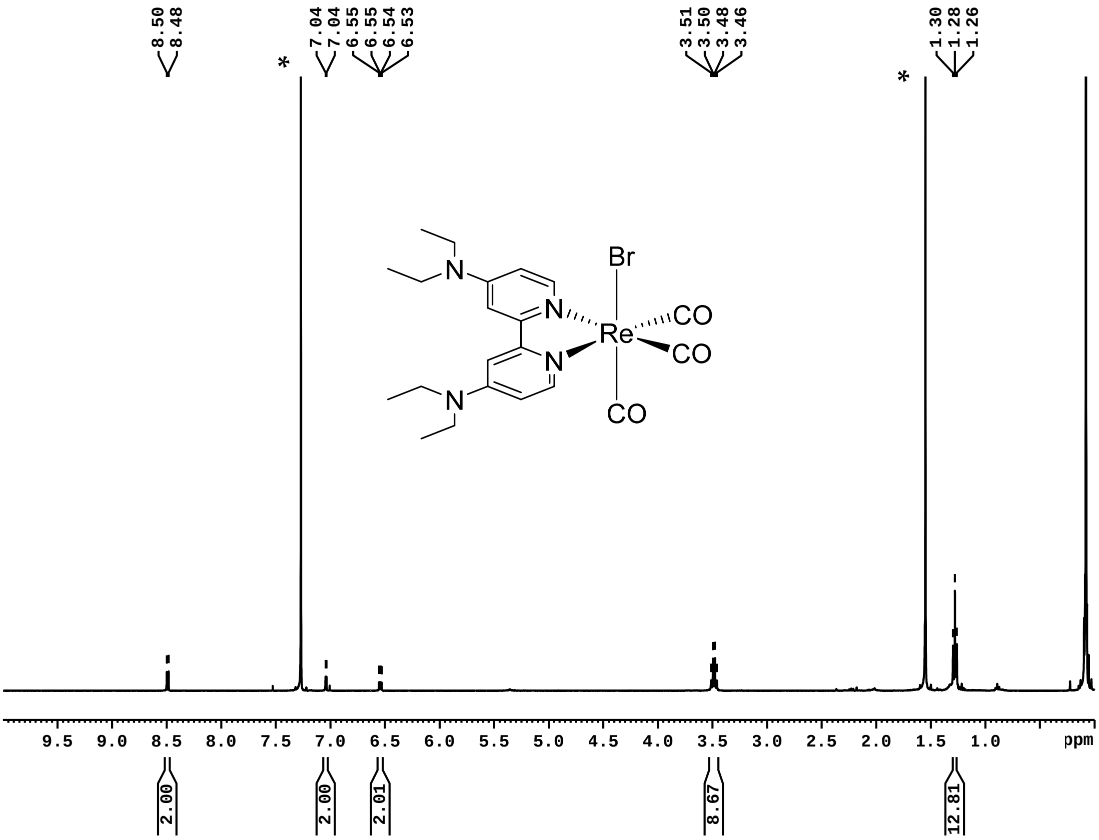

Figure S5. ^{1}H NMR spectrum of **8** in $CDCl_3$, ✱= solvent residual peak ($CHCl_3$, H_2O).

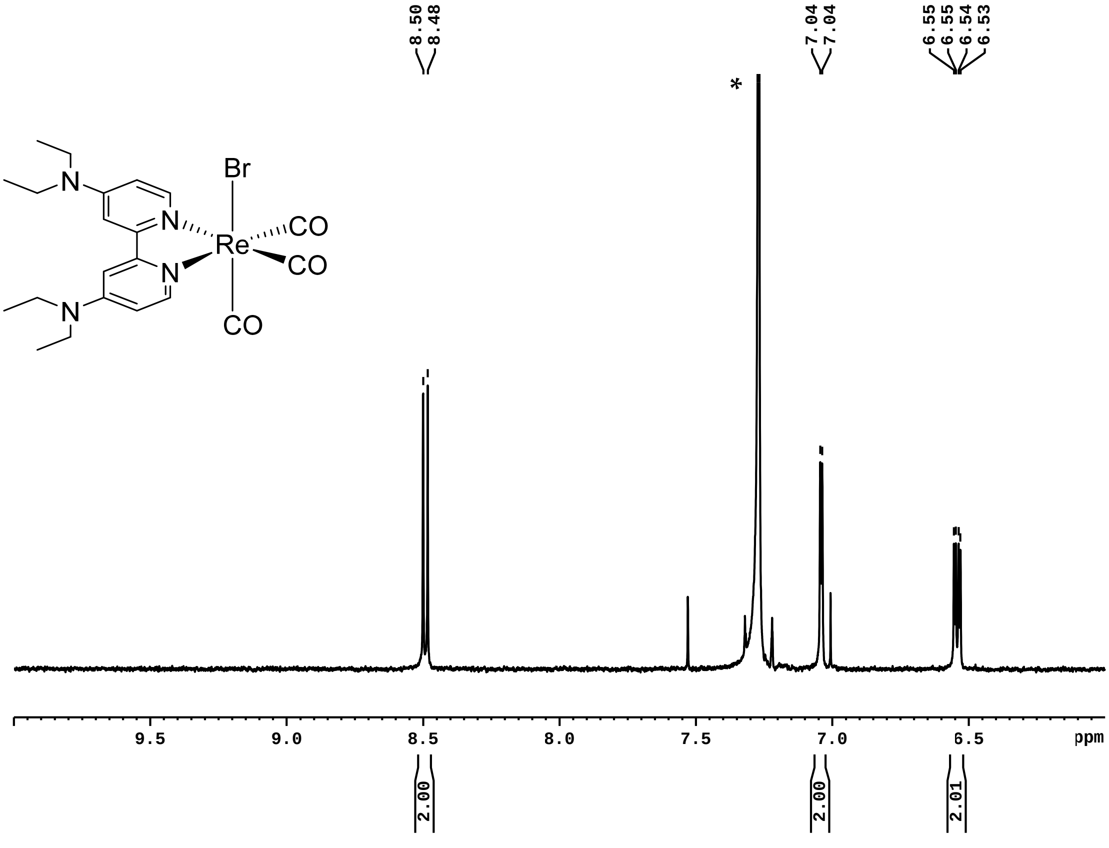

Figure S5a. ^{1}H NMR spectrum of aromatic region of **8** in $CDCl_3$ (zoom), ✱= solvent residual peak ($CHCl_3$).

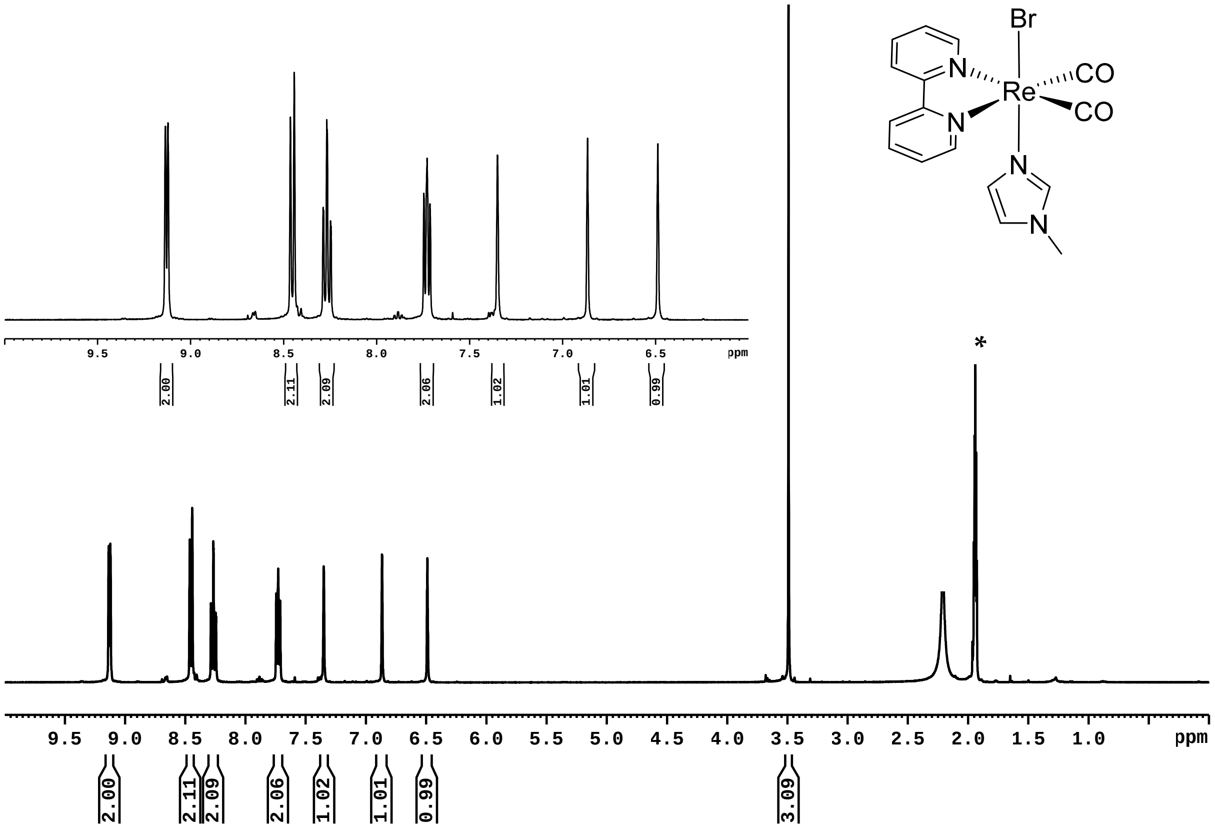

Figure S6. ^{1}H NMR spectrum of **3a** in CD_3CN. ✱= solvent residual peak.

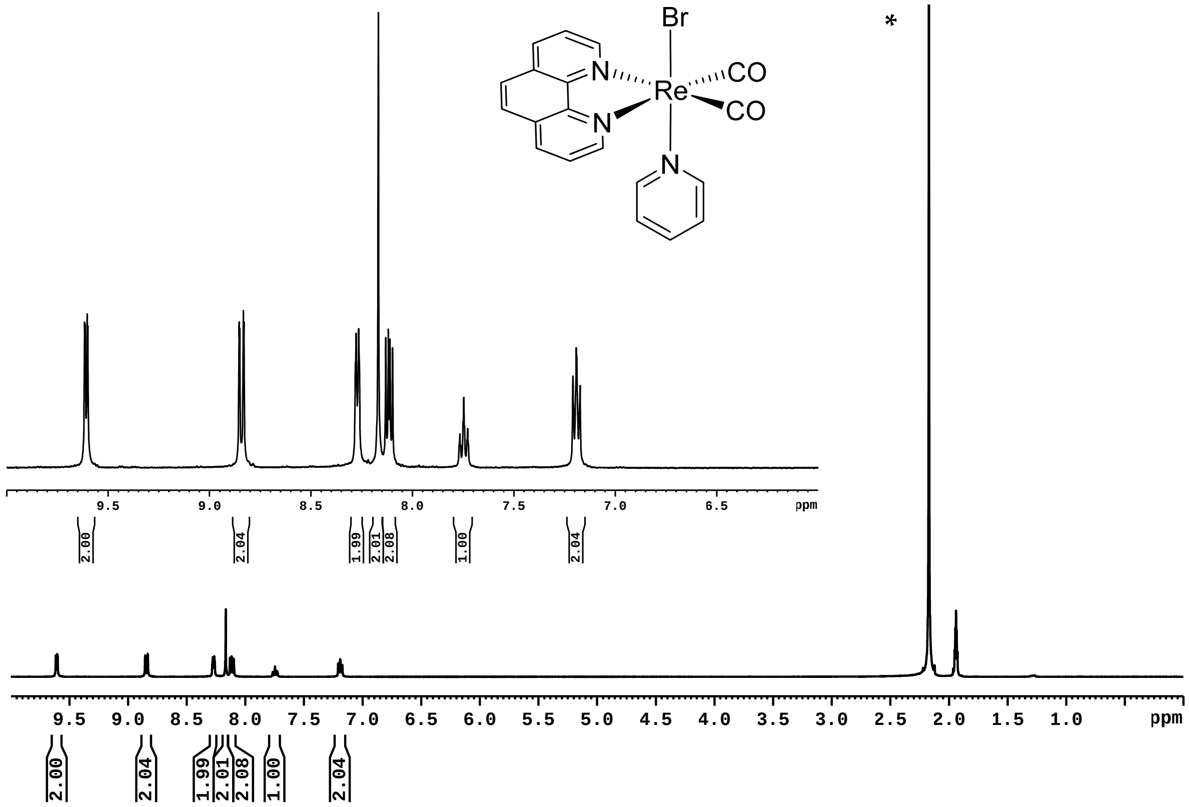

Figure S7. ^{1}H NMR spectrum of **2c** in CD_3CN. ✱= solvent residual peak.

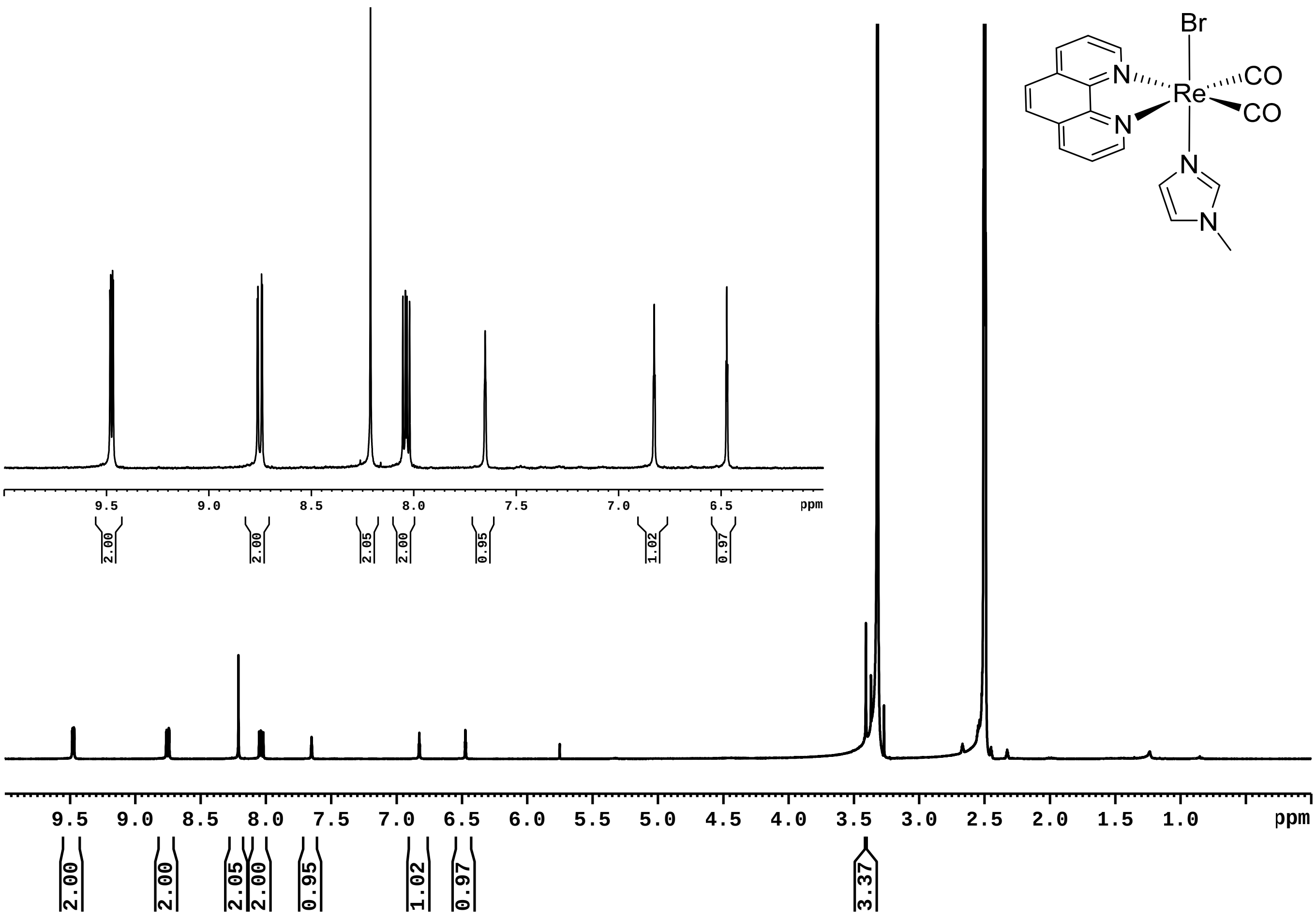

Figure S8. ^{1}H NMR spectrum of **3c** in $(CD_3)_2SO$. ✱= solvent residual peak.

IR spectra

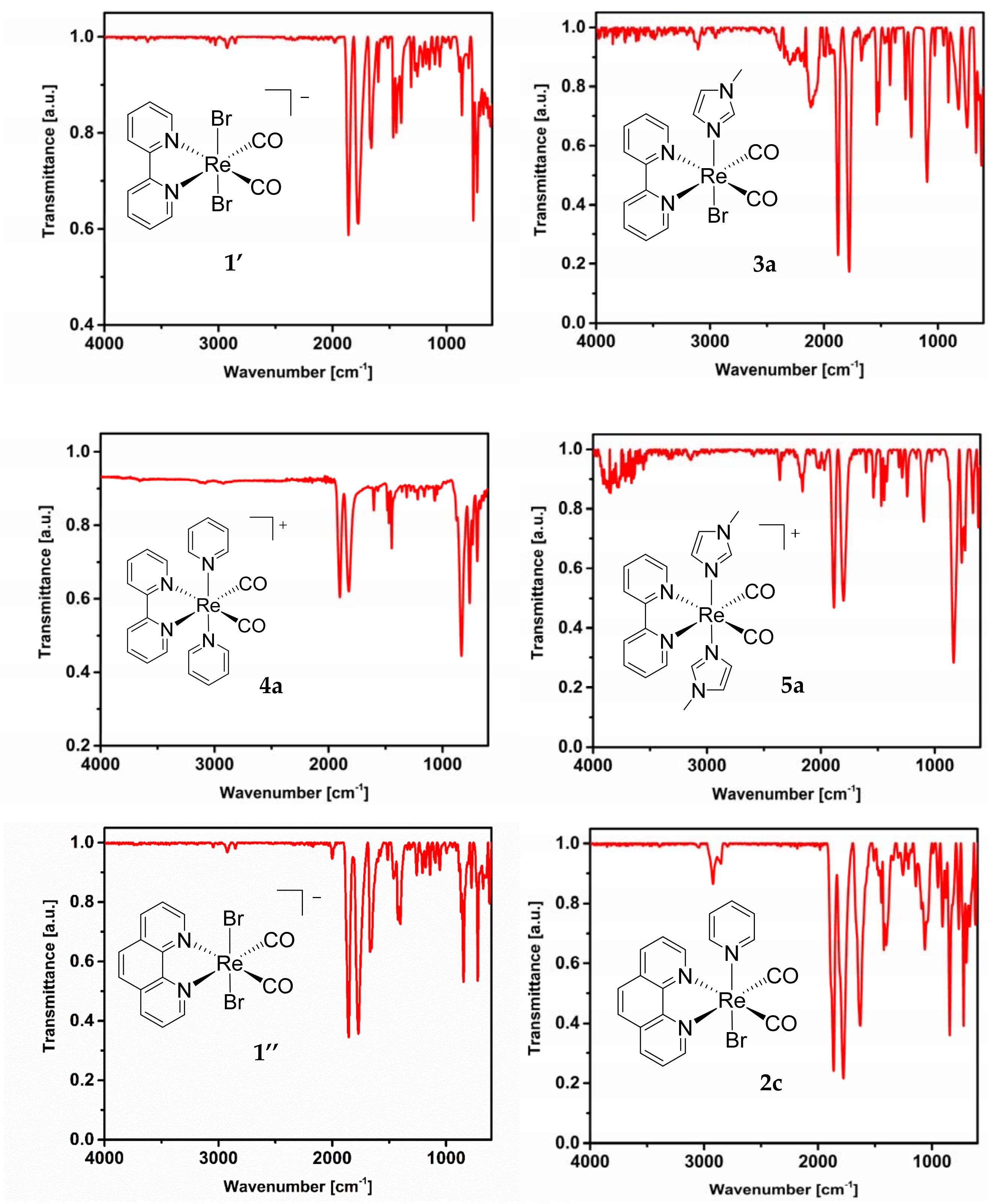

Figure S9. IR spectra (solid) of compounds.

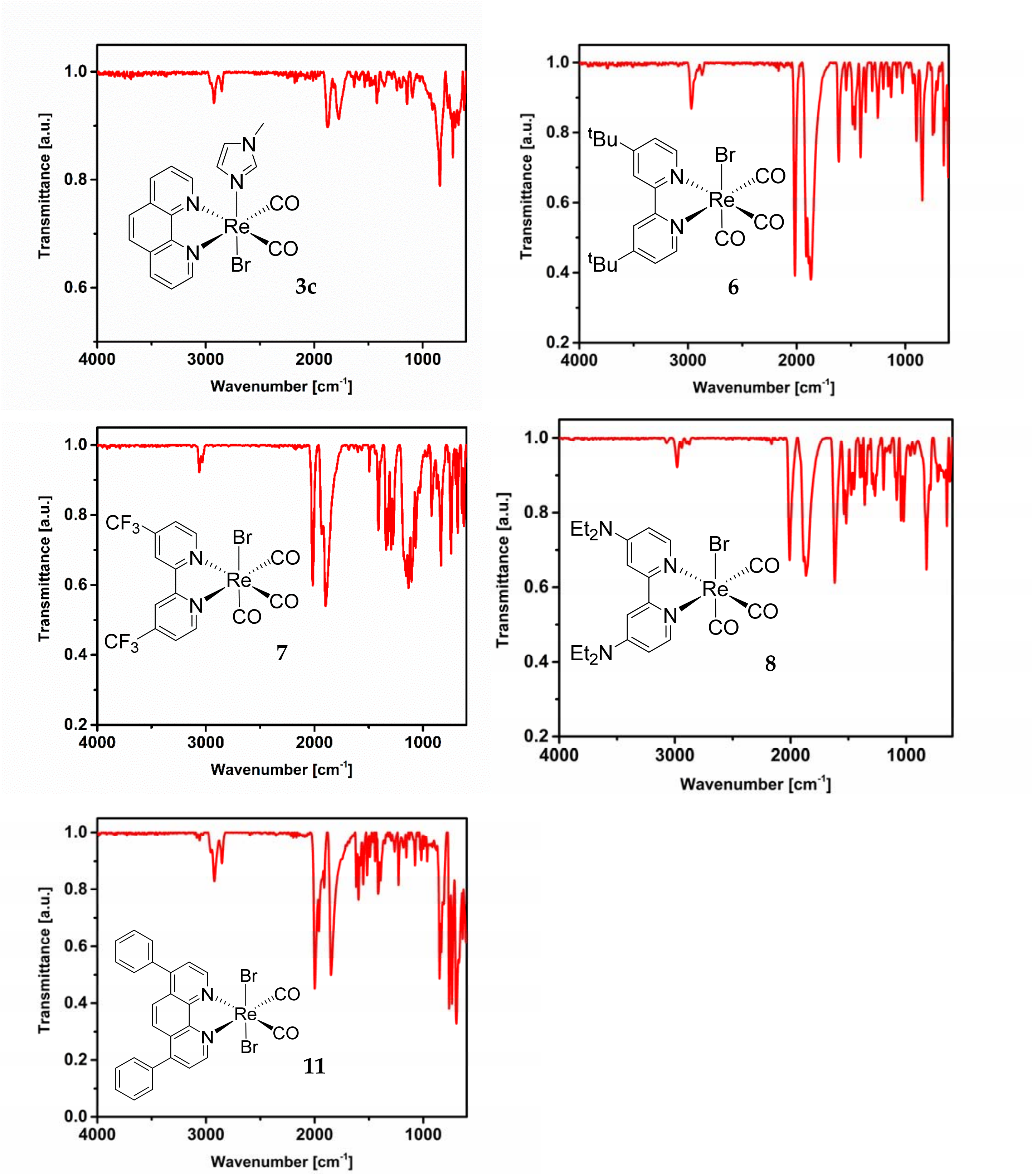

Figure S9. IR spectra (solid) of compounds.

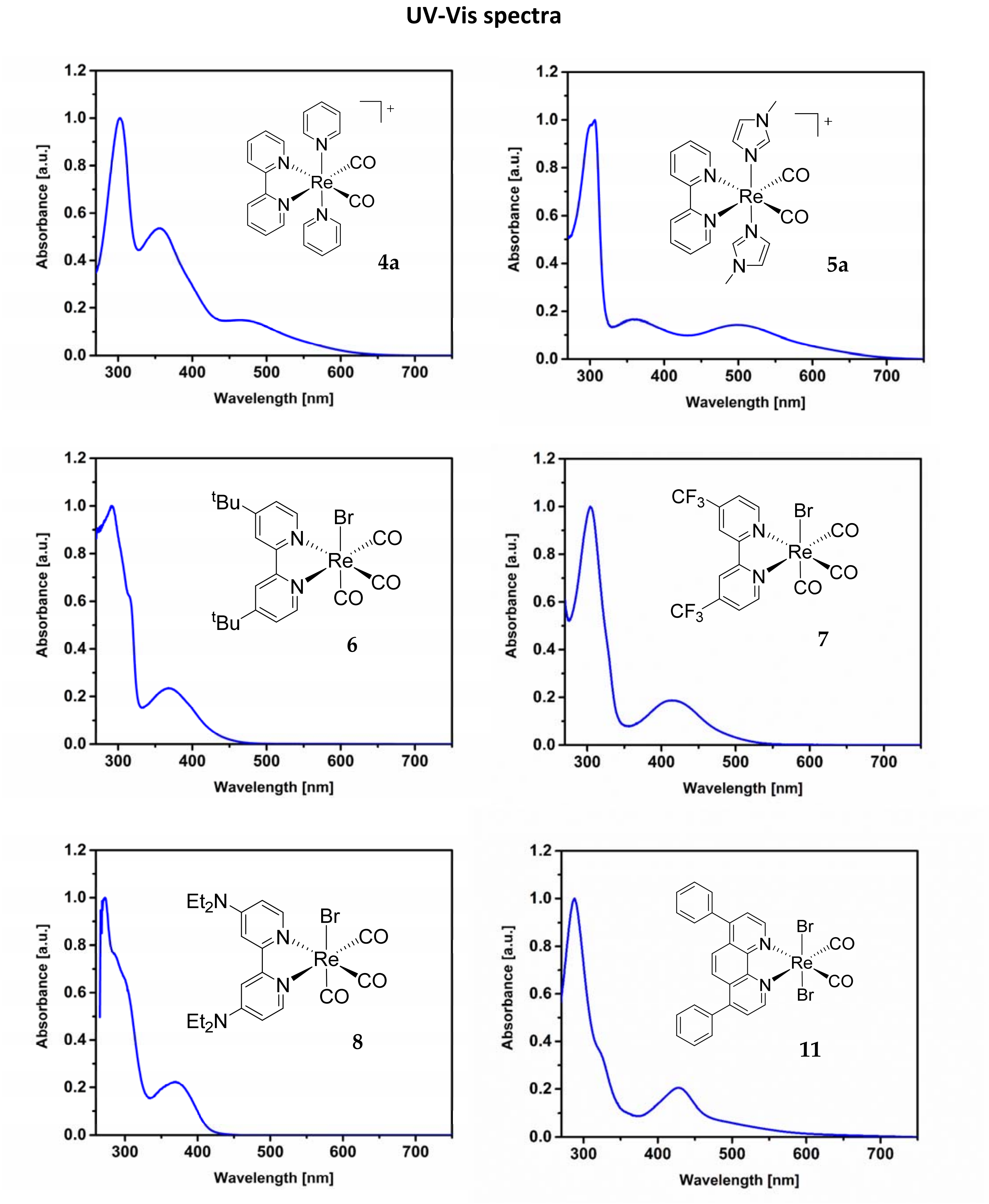

Figure 10. UV-Vis spectrum of compounds (in DMF).

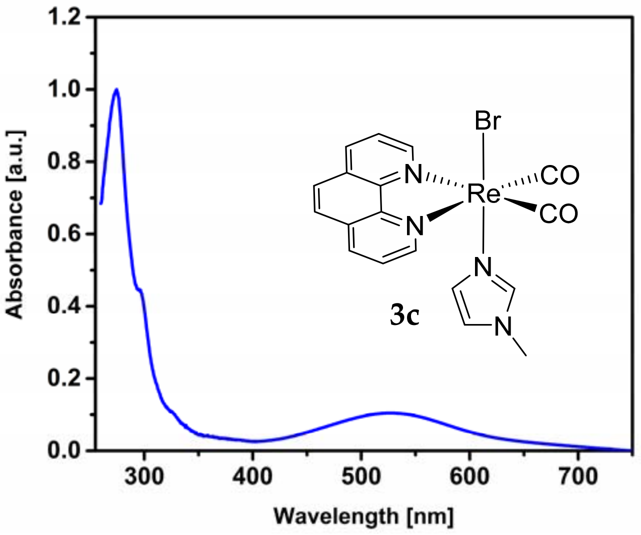

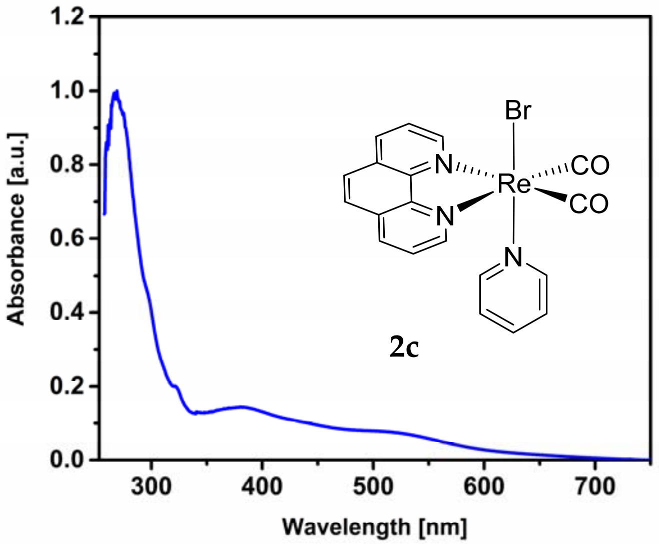

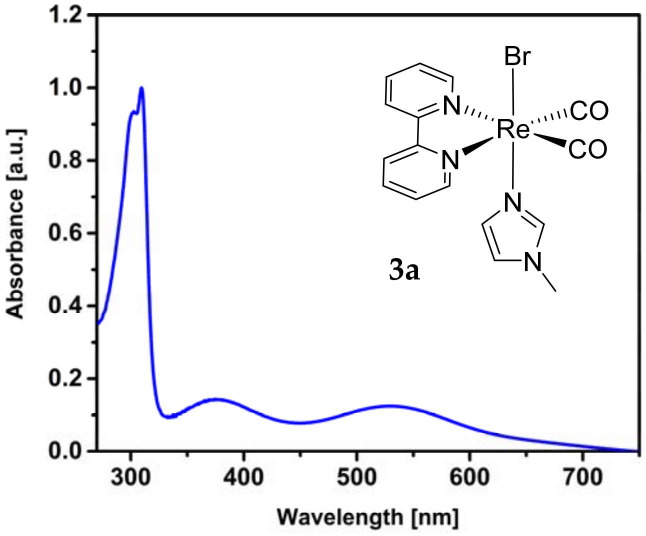

Figure S10 cont'd. UV-Vis spectrum of compounds (in DMF).

Table S1a. *In silico* pre-screening of binding affinities (*b.a.*; docking scores. kcal/mol) of rhenium complexes against structurally characterized membrane bound *S. aureus* proteins: Penicillin Binding Proteins (PBPs).

Complex / Protein	Docking score (kcal/mol)							
	**PBP2**	**Δ**	**PBP2a**	**Δ**	**PBP3**	**Δ**	**PBP4**	**Δ**
Control*	-8.2	0	-9.6	0	-10.5	0	-8.5	0
**1a**	-5.1	+3.4	-6.4	+3.5	-5.7	+5.1	-6.8	+2
**2a**	-6.1	+2.4	-6.2	+3.7	-6.4	+4.4	-8.1	+0.7
**3a**	-6.2	+2.3	-6.0	+3.9	-6.2	+4.6	-8.1	+0.7
**4a**	-6.7	+1.8	-6.3	+3.6	-6.7	+4.1	-6.8	+2
**5a**	-6.4	+2.1	-5.6	+4.3	-6.6	+4.2	-6.5	+2.3
**1b**	-6.4	+2.1	-8.1	+1.8	-7.1	+3.7	**-8.9**	**-0.1**
**2b**	-7.8	+0.7	-6.7	+3.2	-7.4	+3.4	**-9.9**	**-1.1**
**3b**	-7.7	+0.8	-6.6	+3.3	-7.2	+3.6	**-9.6**	**-0.8**
**4b**	-7.9	+0.6	-5.5	+4.4	-6.8	+4	-6.6	+2.2
**5b**	-7.6	+0.9	-5.5	+4.4	-6.7	+4.1	-6.6	+2.2
**1c**	-5.9	+2.6	-7.7	+2.2	-6.7	+4.1	-8.0	+0.8
**2c**	-6.2	+2.3	-6.0	+3.9	-7.0	+3.8	-6.8	+2
**3c**	-6.3	+2.2	-6.2	+3.7	-7.1	+3.7	-8.5	+0.3
**6**	-7.2	+1.3	-8.6	+1.3	-7.7	+3.1	**-9.5**	**-0.7**
**7**	-7.3	+1.2	-8.6	+1.3	-8.6	+2.2	**-9.1**	**-0.3**
**8**	-6.1	+2.4	-7.7	+2.2	-7.4	+3.4	-8.3	+0.5
**9a**	-6.0	+2.5	-8.1	+1.8	-7.0	+3.8	-8.3	+0.5
**9b**	-6.6	+1.9	-6.7	+3.2	-7.1	+3.7	-8.0	+0.8
**9c**	-6.7	+1.8	-7.2	+2.7	-7.2	+3.6	-6.9	+1.9
**10**	-7.9	+0.6	**-10.2**	**-0.3**	**-10.3**	+0.5	**-12.3**	**-3.5**
**13**	-8.1	+0.4	-8.2	+1.7	-8.6	+2.2	**-9.1**	**-0.3**
**14**	-7.6	+0.9	-9.7	+0.2	**-10.0**	+0.8	**-10.2**	**-1.4**
**15**	-8.9	-0.4	-7.3	+2.6	-8.3	+2.5	**-10.5**	**-1.7**
**16**	-6.4	+2.1	-5.9	+4	-7.3	+3.5	-8.5	+0.3
**17**	-9.0	-0.5	-6.8	+3.1	-7.2	+3.6	-8.1	0.7
**18**	-8.3	+0.2	**-9.9**	**0**	-9.4	+1.4	**-10.2**	**-1.4**
**19**	**-9.5**	**-1**	-9.3	+0.6	**-10.5**	+0.3	**-10.7**	**-1.9**

* Ceftobiprole in all cases. Δ refers to: (*b.a.* Re complex + 0.3 kcal/mol)–*b.a.* control. PDB codes: PBP2: 2OLV; PBP2a: 4DKI; PBP3: 3VSL; PBP4: 5TXI.

Table S1b. *In silico* pre-screening of binding affinities (*b.a.*; docking scores. kcal/mol) of non-toxic complexes against other structurally characterized membrane bound *S. aureus* proteins.

Complex / Protein	Docking score (kcal/mol)							
	**eLtaS**	**Δ**	**SpsB**	**Δ**	**LtaA**	**Δ**	**LspA**	**Δ**
Control*	-8.6	0	-8.5	0	-10.2	0	-10.8	0
**1a**	-6.0	+2.9	-5.5	+3.3	-6.2	+4.3	-6.1	+5
**2a**	-6.8	+2.1	-6.2	+2.6	-7.1	+3.4	-7.1	+4
**3a**	-7.0	+1.9	-6.2	+2.6	-6.9	+3.6	-7.1	+4
**4a**	-7.2	+1.7	-6.1	+2.7	-7.6	+2.9	-7.1	+4
**5a**	-7.2	+1.7	-5.9	+2.9	-6.9	+3.6	-7.1	+4
**1b**	-6.8	+2.1	-6.2	+2.6	-7.6	+2.9	-7.4	+3.7
**2b**	-7.5	+1.4	-6.7	+2.1	-8.5	+2	-7.7	+3.4
**3b**	-7.2	+1.7	-6.4	+2.4	-8.2	+2.3	-7.3	+3.8
**4b**	-7.2	+1.7	-6.6	+2.2	-8.1	+2.4	-7.1	+4
**5b**	-7.0	+1.9	-6.5	+2.3	-7.6	+2.9	-7.0	+4.1
**1c**	-7.1	+1.8	-7.4	+1.4	-7.2	+3.3	-7.3	+3.8
**2c**	-7.1	+1.8	-6.4	+2.4	-7.7	+2.8	-7.4	+3.7
**3c**	-7.3	+1.6	-7.0	+1.8	-7.9	+2.6	-7.3	+3.8
**6**	-7.0	+1.9	-6.5	+2.3	-8.3	+2.2	-7.5	+3.6
**7**	-7.9	+1	-6.9	+1.9	-8.1	+2.4	-8.2	+2.9
**8**	-5.7	+3.2	-5.6	+3.2	-7.6	+2.9	-6.9	+4.2
**9a**	-7.0	+1.9	-7.0	+1.8	-7.3	+3.2	-6.6	+4.5
**9b**	-6.9	+2	-6.2	+2.6	-7.3	+3.2	-7.3	+3.8
**9c**	-7.1	+1.8	-7.2	+1.6	-7.7	+2.8	-7.1	+4
**10**	-8.4	+0.5	-7.7	+1.1	**-10.3**	**+0.2**	-9.3	+1.8
**13**	-7.9	+1	**-9.1**	**-0.3**	-9.2	+1.3	-8.9	+2.2
**14**	-8.8	+0.1	-8.0	+0.8	-9.7	+0.8	-9.7	+1.4
**15**	**-9.4**	**-0.5**	**-9.6**	**-0.8**	**-10.4**	**+0.1**	-8.7	+2.4
**16**	-6.5	+2.4	-7.5	+1.3	-7.9	+2.6	-7.0	+4.1
**17**	-8.9	0	-7.4	-1.1	-9.4	+1.1	-8.9	+2.2
**18**	**-10.8**	**-1.9**	**-9.8**	**-1**	**-10.5**	**0**	**-10.6**	+0.5
**19**	**-10.9**	**-2**	**-10.4**	**-1.6**	**-11.3**	**-0.8**	-9.3	+1.8

* Controls are respectively: compound 1771 (eLtaS and LtaA) [1]; Arilomycin A2 (SpsB) [2]; myxovirescin (LspA) [3]. Δ refers to: b.a. control–(b.a. Re complex + 0.3 kcal/mol). PDB codes: Lipoteichoic acids synthase (eLtaS): 2W5Q; Type-I signal peptidase (SpsB): 4WVJ; Lipoteichoic acids flippase (LtaA): 6S7V; Lipoprotein signal peptidase II (LspA): 6RYP.

Protein surface visualisations of the non-polar polar ratio (NPP) and patch analisys for thr electrostatic surface potential. The protein-sol server (https://protein-sol.manchester.ac.uk/) [4] for sequence-based prediction of protein solubility was used for for calculating and mapping patches of hydrophobicity and charge on the protein surface.

The large hydrophobic surface (higher NPP ratio) colored in green is for the case of **3VSL** and **5TXI** presened in Figure S8, and **6S7V and 6RYP** presented in Figure S9**.** Patch analysis is introduced for electrostatic potential, using the Finite Difference Poisson-Boltzmann (FDPB) methods [5] that aids visualisation of asymmetric charge distributions.

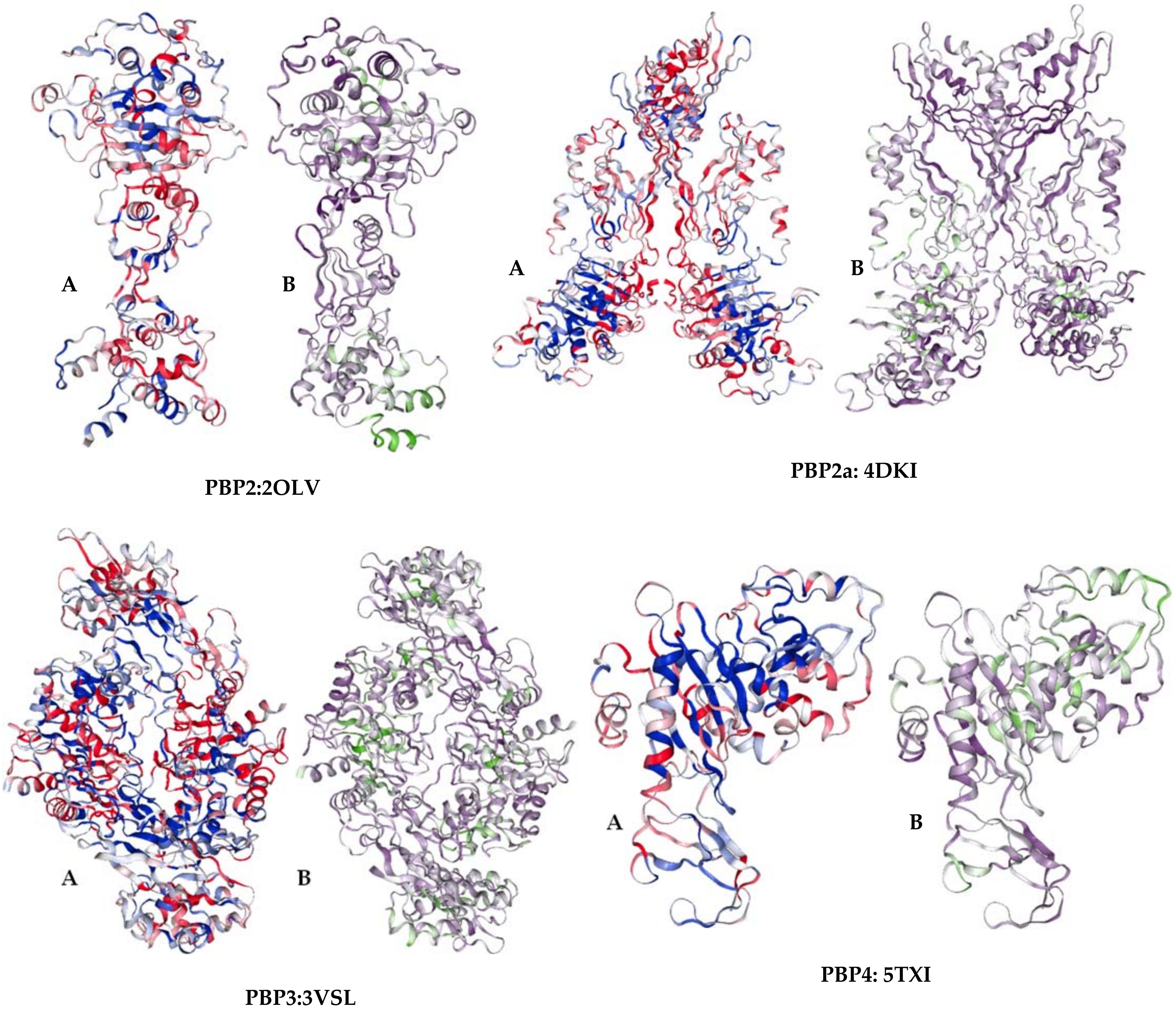

Figure S11. Visualisation of surface protein surface polarity (**A**) non-polar to polar SASA colour-coded from low NPP ratio (purple) to high NPP ratio (green), and in (**B**) colour-coded from negative charge (red) to positive charge (blue). Regions of high hydrophobicity are coloured green, low hydrophobicity coloured purple.

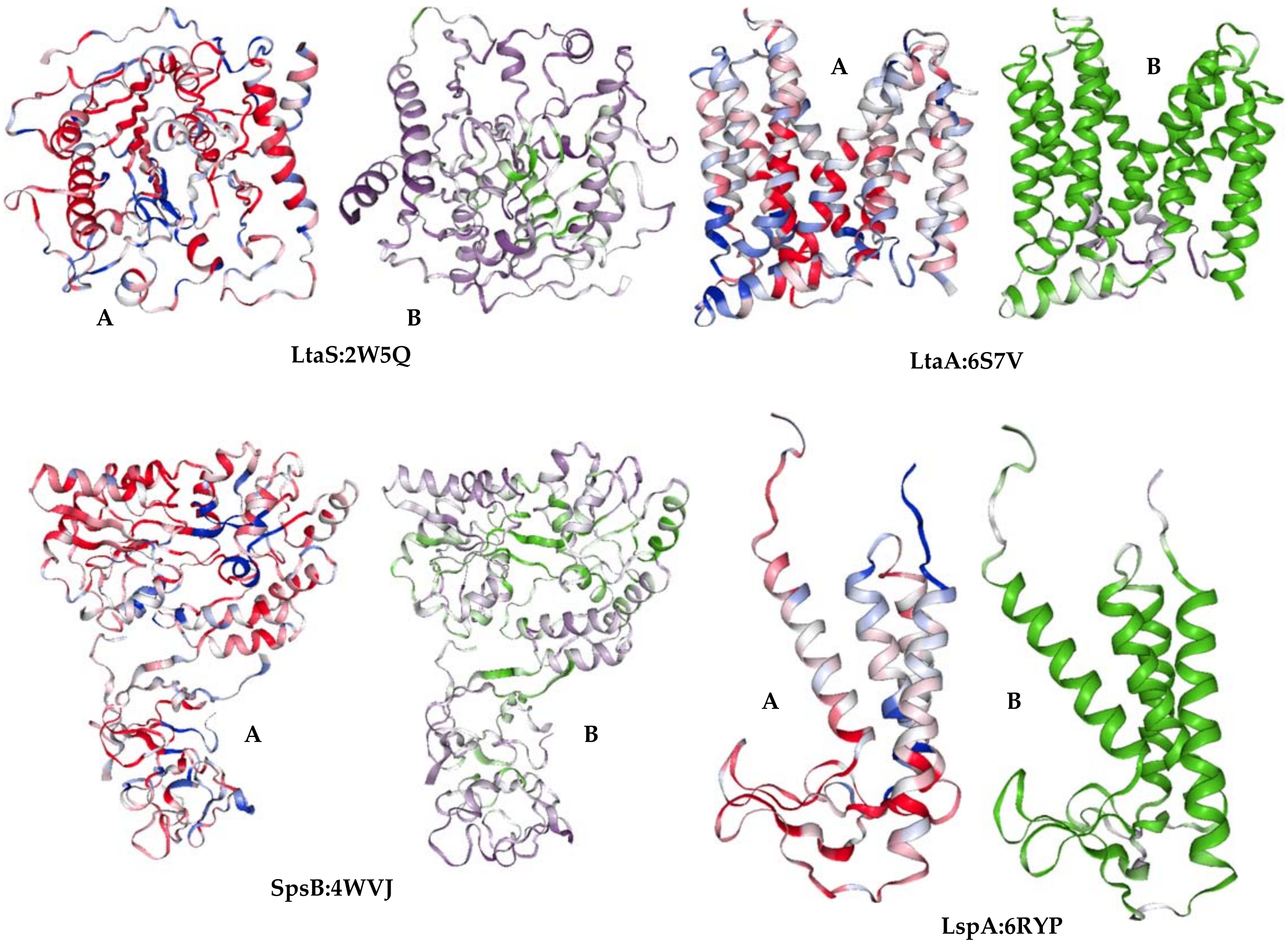

Figure S11. Visualisation of surface protein surface polarity (**A**) non-polar to polar SASA colour-coded from low NPP ratio (purple) to high NPP ratio (green), and in (**B**) colour-coded from negative charge (red) to positive charge (blue). Regions of high hydrophobicity are coloured green, low hydrophobicity coloured purple.

Table S2. Pockets prediction–mapping the ranking with residues environment distribution

**2OLV**	rank	score	probability	sas_points	surf_atoms	residue_ids
pocket1/blue	1	9.11	0.534	120	49	B_114 B_124 B_129 B_132 B_133 B_145 B_146 B_147 B_151 B_152 B_155 B_156 B_165 B_167 B_168 B_171 B_195 B_196 B_231 B_232
pocket2/red	2	8.43	0.496	109	52	A_114 A_129 A_145 A_146 A_147 A_151 A_152 A_155 A_156 A_163 A_165 A_167 A_168 A_171 A_195 A_196 A_231 A_232
pocket3	3	7.11	0.406	67	31	B_398 B_435 B_436 B_437 B_453 B_454 B_455 B_456 B_496 B_569 B_584 B_586 B_587 B_588 B_641

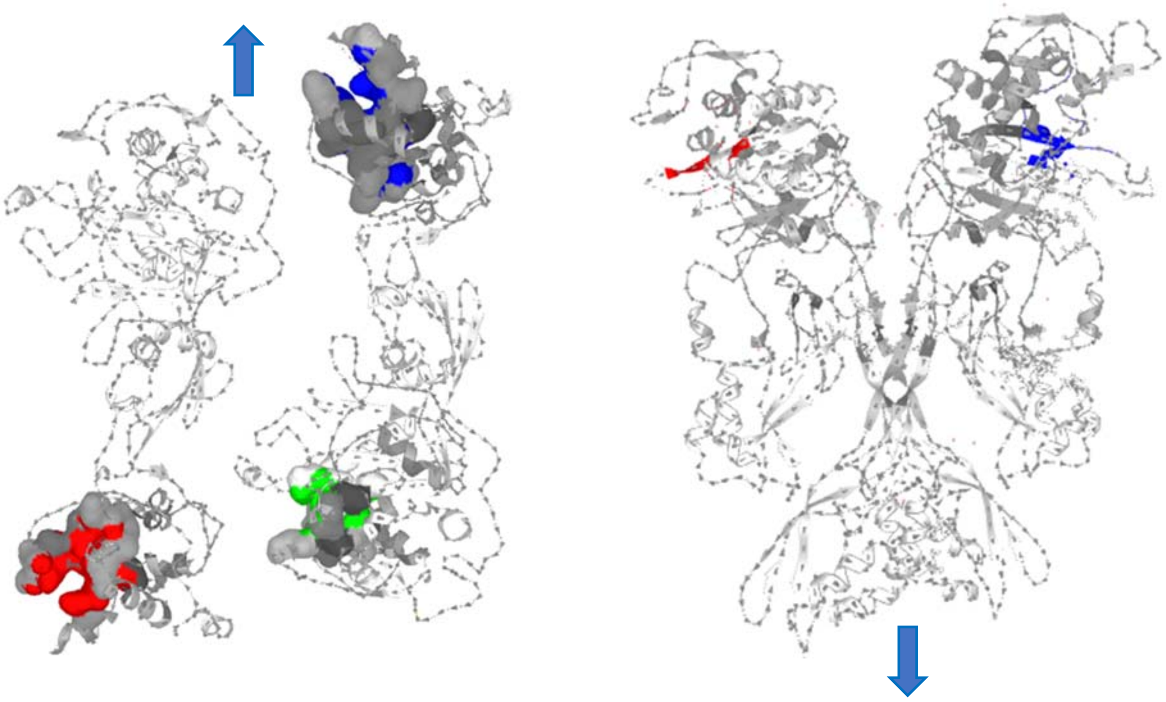

**4DKI**	rank	score	robability	sas_points	surf_atoms	residue_ids
pocket1/blue	1	20.2	0.84	143	67	A_399 A_400 A_402 A_403 A_444 A_446 A_447 A_461 A_462 A_464 A_520 A_521 A_522 A_582 A_583 A_598 A_599 A_600 A_601 A_602 A_605 A_614 A_616 A_641 A_642 A_643
pocket2/red	2	10.15	0.587	98	47	B_400 B_402 B_403 B_444 B_446 B_461 B_462 B_464 B_519 B_520 B_521 B_522 B_582 B_583 B_598 B_600 B_601 B_602 B_614 B_642

**3VSL**	rank	score	probability	sas_points	surf_atoms	residue_ids
pocket1/blue	1	10.85	0.618	131	55	A_392 A_426 A_427 A_429 A_447 A_448 A_450 A_454 A_519 A_522 A_524 A_603 A_604 A_606 A_607 A_619 A_621 A_623 A_632 A_634 A_636 A_656 A_658 A_660 A_663 A_664
pocket2 /red	2	9.19	0.538	68	42	B_392 B_428 B_429 B_430 B_447 B_448 B_450 B_454 B_522 B_524 B_603 B_619 B_620 B_621 B_622 B_623 B_659 B_661

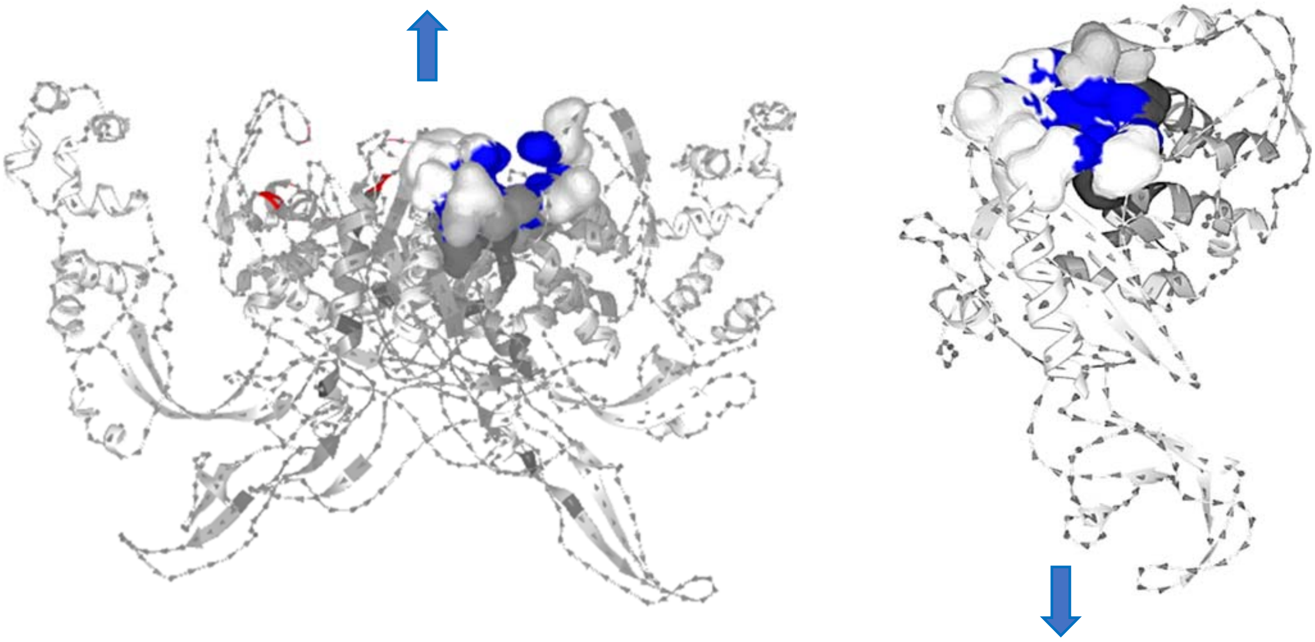

**5TXI**	rank	score	probability	sas_points	surf_atoms	residue_ids
pocket1	1	15.7	0.763	76	37	A_114 A_115 A_139 A_141 A_180 A_181 A_182 A_183 A_186 A_241 A_260 A_262 A_263 A_264 A_268 A_291 A_297 A_75

**4WVJ**	rank	score	probability	sas_points	surf_atoms	residue_ids	surf_atom_ids
pocket1/blue	1	17.85	0.808	107	62	A_117 A_156 A_159 A_161 A_162 A_18 A_20 A_21 A_216 A_236 A_268 A_343 A_346 A_347 A_350 A_48 A_50 A_51 A_67 A_68 A_69 A_71 A_72	46 50 51 52 53 62 65 74 274 279 282 297 298 299 308 407 422 423 424 426 429 433 450 451 459 460 812 1117 1140 1141 1142 1143 1155 1156 1157 1158 1159 1160 1161 1171 1173 1579 1580 1581 1582 1731 1732 1976 2538 2559 2560 2561 2562 2563 2564 2565 2566 2567 2568 2571 2604 2608
pocket2 /red	2	3.16	0.11	28	21	A_116 A_265 A_267 A_327 A_330 A_331 A_335 A_98	653 654 655 656 657 658 659 803 1956 1957 1958 1969 2418 2438 2439 2440 2441 2472 2476 2477 2479

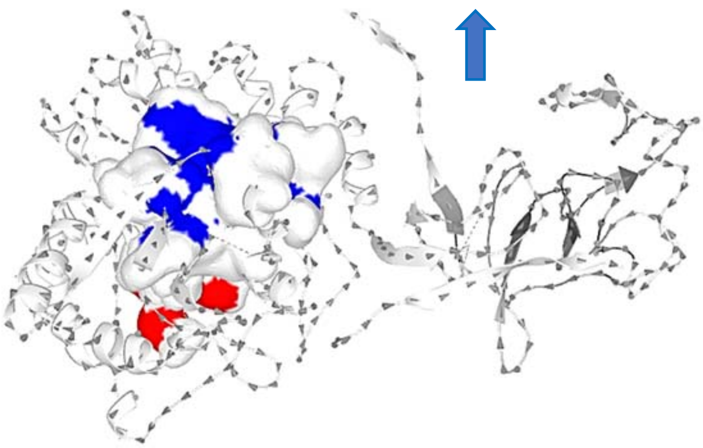

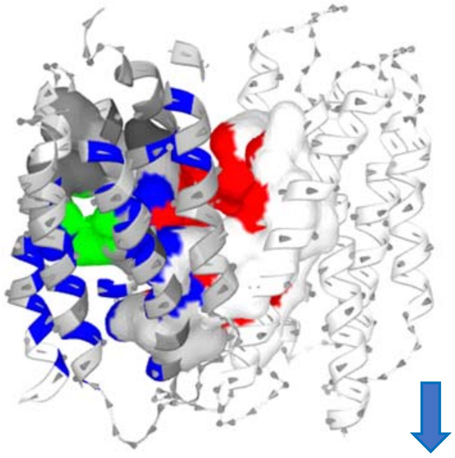

**6S7V**	rank	score	probability	surf_atoms	residue_ids
pocket1 /blue	1	27.34	0.901	96	A_219 A_221 A_222 A_225 A_226 A_229 A_230 A_233 A_234 A_237 A_240 A_256 A_259 A_260 A_263 A_283 A_286 A_287 A_290 A_293 A_300 A_309 A_312 A_313 A_316 A_320 A_323 A_324 A_327 A_330 A_377 A_380 A_381 A_383 A_384 A_387 A_388 A_391
pocket2 /red	2	21.7	0.855	58	A_123 A_127 A_131 A_147 A_150 A_154 A_230 A_231 A_32 A_320 A_324 A_327 A_328 A_348 A_35 A_351 A_352 A_355 A_356 A_359 A_40 A_61 A_65 A_68 A_72
pocket3/green	3	5.91	0.31	20	A_224 A_225 A_228 A_229 A_357 A_361 A_365 A_376 A_380

**6RYP**	rank	score	probability	surf_atoms	residue_ids
pocket1/blue	1	28.61	0.909	85	A_103 A_106 A_107 A_110 A_116 A_118 A_125 A_129 A_130 A_131 A_132 A_136 A_137 A_139 A_140 A_143 A_50 A_51 A_52 A_53 A_54 A_55 A_56 A_57 A_58 A_59 A_67 A_70 A_71 A_74
pocket2/red	2	6.18	0.333	39	A_10 A_100 A_104 A_11 A_14 A_15 A_18 A_7 A_79 A_82 A_83 A_96 A_97

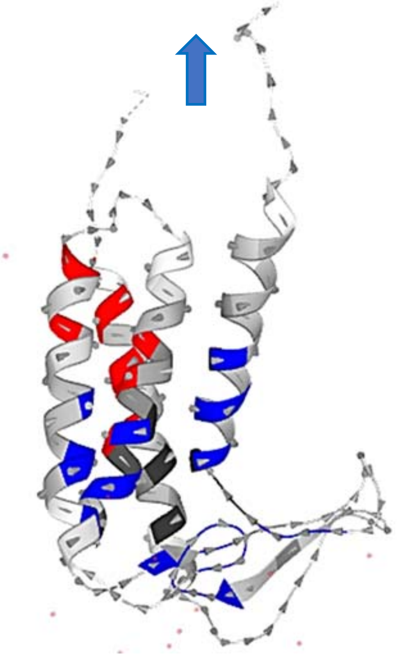

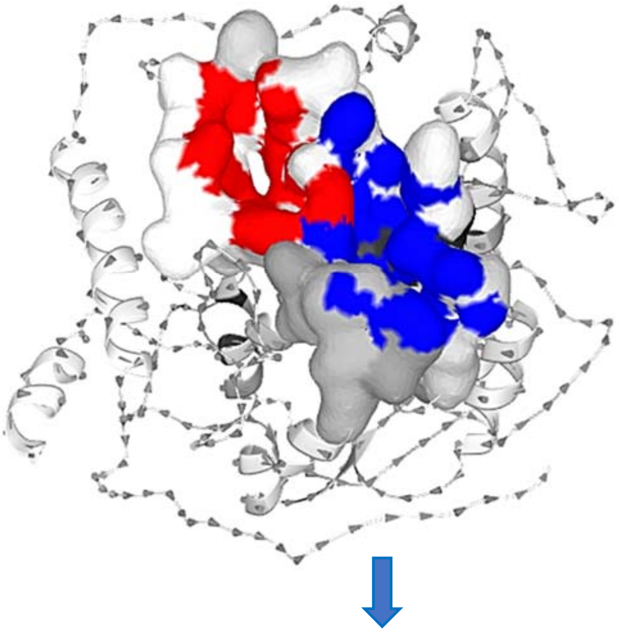

**2W5Q**	rank	score	probability	sas_points	surf_atoms	residue_ids
pocket1	1	25.63	0.888	95	41	A_255 A_256 A_298 A_299 A_300 A_301 A_347 A_349 A_353 A_354 A_356 A_384 A_413 A_416 A_417 A_476 A_477 A_478 A_480
pocket2	2	6.26	0.338	65	36	A_293 A_294 A_295 A_296 A_299 A_477 A_481 A_497 A_500 A_501 A_504 A_562 A_563 A_564 A_566 A_575

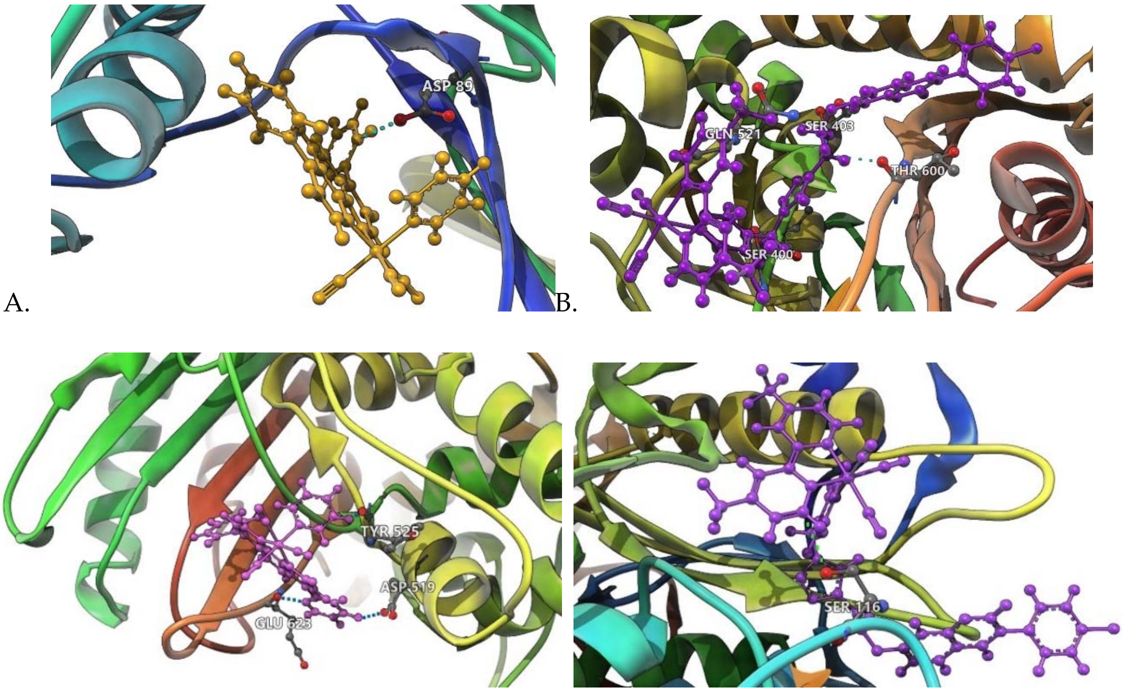

A. B. C. D.

Figure S12a. Binding orientation of the compounds with hydrogen-acceptor and hydrogen-donor distances: A. **15** and PBP2: 2OLV ; B. **19** and PBP2a: 4DKI ; C. **15** and PBP3: 3VSL; D. **19** and PBP4: 5TXI.

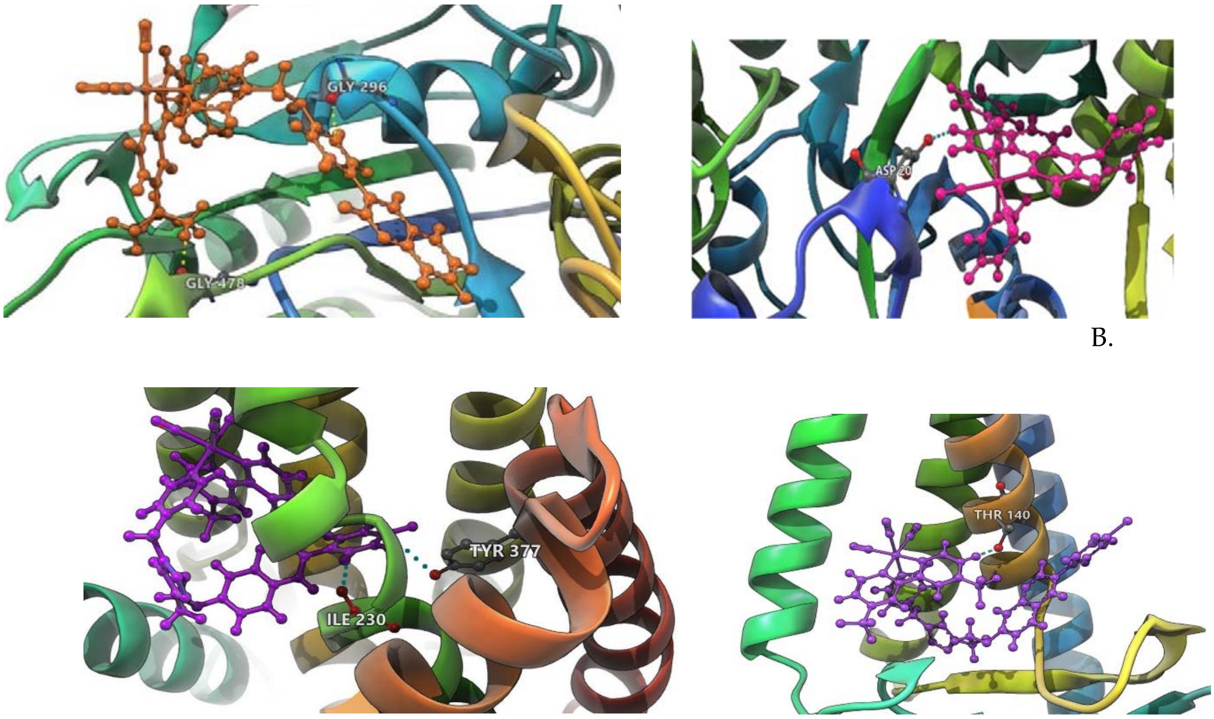

A. B. C. D

Figure S12b. Binding orientation of the compounds with hydrogen-acceptor and hydrogen-donor distances: A. **18** and Lipoteichoic acids synthase (LtaS): 2W5Q; B. **15** and Type-I signal peptidase (SpsB): 4WVJ; C. **19** and Lipoteichoic acids flippase (LtaA): 6S7V; D. **19** and Lipoprotein signal peptidase II (LspA): 6RYP.

Table S3. Percentage distribution of the surrounding residue types for the two groups of protein.

2OLV	Amini Acid	Number	Percentage	4DKI	Amini Acid	Number	Percentage	3VSL	Amini Acid	Number	Percentage	5TXI	Type	Number	Percentage
	LYS	4	17,3913		SER	6	20.0000		GLN	4	20.0000		SER	6	23,0769
	ASP	3	13,0435		GLY	4	13.3333		ASN	3	15.0000		THR	4	15,3846
	GLN	2	8,6957		GLU	3	10.0000		ASP	2	10.0000		GLU	3	11,5385
	LEU	2	8,6957		TYR	3	10.0000		GLU	2	10.0000		TYR	3	11,5385
	GLU	1	4,3478		LYS	3	10.0000		THR	2	10.0000		PHE	2	7,6923
	GLY	1	4,3478		GLN	2	6.6667		PHE	2	10.0000		ALA	1	3,8462
	HIS	2	8,6957		ALA	2	6.6667		ALA	1	5.0000		ARG	1	3,8462
	VAL	2	8,6957		THR	2	6.6667		VAL	1	5.0000		ASP	1	3,8462
	ILE	1	4,3478		ASP	1	3.3333		ILE	1	5.0000		ASN	1	3,8462
	PRO	1	4,3478		ASN	1	3.3333		TYR	1	5.0000				
	THR	1	4,3478		HIS	1	3.3333						VAL	1	3,8462
	TYR	1	4,3478		LEU	1	3.3333						GLY	1	3,8462
	ALA	1	4,3478		MET	1	3.3333						LEU	1	3,8462
	ARG	1	4,3478										LYS	1	3,8462

2W5Q	Type	Number	Percentage	4WVJ	Type	Number	Percentage	6S7V	Type	Number	Percentage	6RYP	Type	Number	Percentage
	THR	4	11,7647		TYR	3	16,6667		LEU	6	22.2222		ASN	3	13,6364
	PHE	4	11,7647		ASP	2	11,1111		ALA	4	14.8148		ILE	3	13,6364
	HIS	4	11,7647		ASN	2	11,1111		ILE	4	14.8148		PHE	3	13,6364
	SER	3	8,8235		ARG	1	5,5556		GLY	3	11.1111		ALA	2	9,0909
	GLY	3	8,8235		HIS	1	5,5556		MET	3	11.1111		ASP	2	9,0909
	ASN	3	8,8235		GLY	1	5,5556		TYR	3	11.1111		LEU	2	9,0909
	TYR	2	5,8824		GLU	1	5,5556		VAL	1	3.7037		ARG	1	4,5455
	GLU	2	5,8824		GLN	1	5,5556		PRO	1	3.7037		VAL	1	4,5455
	GLN	2	5,8824		LEU	1	5,5556		TRP	1	3.7037		GLY	1	4,5455
	ASP	2	5,8824		LYS	1	5,5556		CYS	1	3.7037		PRO	1	4,5455
	TRP	1	2,9412		PRO	1	5,5556						SER	1	4,5455
	PRO	1	2,9412		SER	1	5,5556						THR	1	4,5455
	LYS	1	2,9412		TRP	1	5,5556						TRP	1	4,5455
	LEU	1	2,9412		PHE	1	5,5556								
	ILE	1	2,9412												

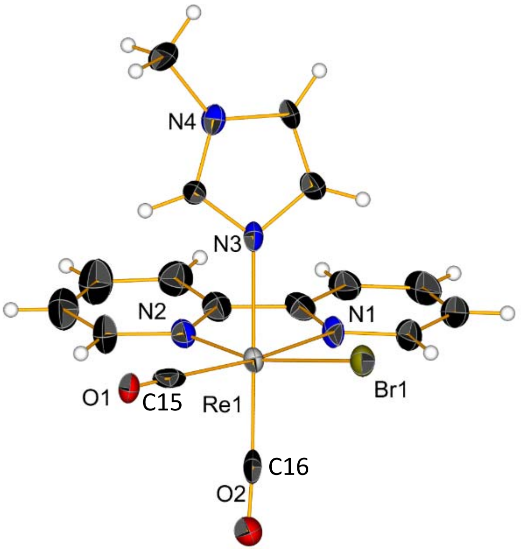

Table S4a Selected Bond Lengths for ***cis*-3a**.

Atom	Atom	Length/Å
Re1	Br1	2.606(2)
Re1	N1	2.197(10)
Re1	N2	2.103(12)
Re1	N3	2.188(14)
Re1	C15	2.00(2)
Re1	C16	1.902(19)
O1	C15	0.93(2)
O2	C16	1.15(2)

Table S4a′ Selected Bond Angles for ***cis*-3a**.

Atom	Atom	Atom	Angle/°	Atom	Atom	Atom	Angle/°	Atom	Atom	Atom	Angle/°
N1	Re1	Br1	91.8(3)	N2	Re1	Br1	163.9(3)	C16	Re1	Br1	96.5(5)
N2	Re1	N1	75.6(4)	N2	Re1	N3	83.1(5)	C16	Re1	N1	96.1(6)
N3	Re1	Br1	85.4(3)	N3	Re1	N1	82.6(4)	C16	Re1	N2	94.8(6)
C15	Re1	Br1	96.0(4)	C15	Re1	N1	171.3(5)	C16	Re1	N3	177.8(7)
C15	Re1	N2	96.0(5)	C15	Re1	N3	94.3(5)	C16	Re1	C15	86.7(6)

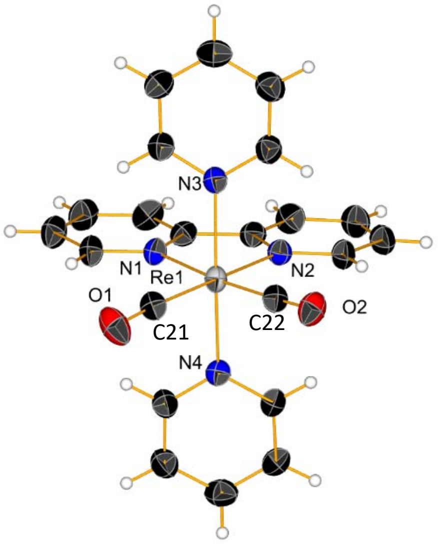

Table S4b Selected Bond Lengths for **4a**.

Atom	Atom	Length/Å
Re1	N1	2.181(5)
Re1	N2	2.183(5)
Re1	N3	2.175(5)
Re1	N4	2.165(5)
Re1	C21	1.876(7)
Re1	C22	1.880(7)
O1	C21	1.174(8)
O2	C22	1.169(8)

Table S4b′ Selected Bond Angles for **4a**.

Atom	Atom	Atom	Angle/°	Atom	Atom	Atom	Angle/°	Atom	Atom	Atom	Angle/°
N1	Re1	N2	74.26(19)	N3	Re1	N1	86.65(19)	C21	Re1	N4	90.1(2)
N3	Re1	N2	87.05(19)	N4	Re1	N2	85.66(19)	C22	Re1	N3	91.4(2)
N4	Re1	N1	87.12(19)	N4	Re1	N3	171.46(19)	C22	Re1	N1	173.8(2)
C21	Re1	N1	99.1(2)	C21	Re1	N3	96.6(2)	C22	Re1	N4	94.2(2)
C21	Re1	N2	172.2(2)	C21	Re1	C22	87.1(3)	C22	Re1	N2	99.7(2)

Table S4c Selected Bond Lengths for **11**.

Atom	Atom	Length/Å
Re1	Br1	2.5117(5)
Re1	Br2	2.5199(4)
Re1	N1	2.154(3)
Re1	N2	2.150(3)
Re1	C25	1.987(5)
Re1	C26	1.957(5)
O1	C25	1.087(5)
O2	C26	1.077(5)

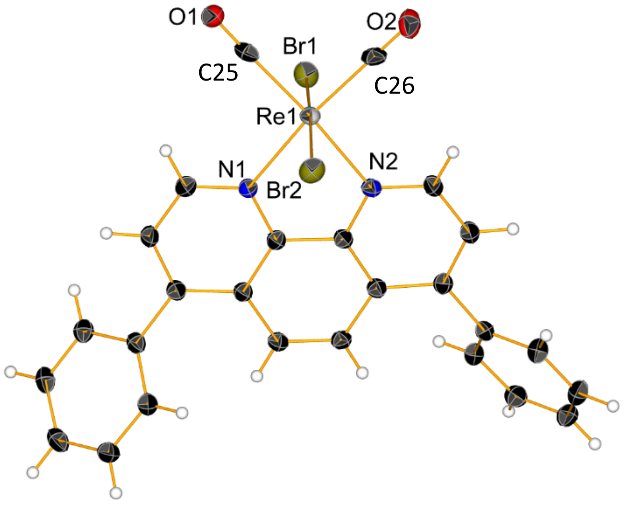

Table S4c′ Selected Bond Angles for **11**.

Atom	Atom	Atom	Angle/°	Atom	Atom	Atom	Angle/°	Atom	Atom	Atom	Angle/°
Br1	Re1	Br2	172.749(18)	N1	Re1	Br1	86.11(8)	C26	Re1	Br1	94.61(13)
N1	Re1	Br2	87.63(8)	N2	Re1	Br1	87.62(8)	C26	Re1	Br2	91.21(13)
N2	Re1	Br2	87.29(8)	N2	Re1	N1	75.69(11)	C26	Re1	N1	173.63(14)
C25	Re1	Br1	88.49(12)	C25	Re1	Br2	96.09(12)	C26	Re1	N2	98.00(14)
C25	Re1	N1	98.86(14)	C25	Re1	N2	173.51(14)	C26	Re1	C25	87.49(16)

Table S4d Selected Bond Lengths for **6**.

Atom	Atom	Length/Å
Re1	Br1	2.6115(7)
Re1	N1	2.169(4)
Re1	N2	2.177(5)
Re1	C19	1.940(6)
Re1	C20	1.929(5)
Re1	C21	1.986(7)
O1	C19	1.125(7)
O2	C20	1.138(7)
O3	C21	1.061(7)

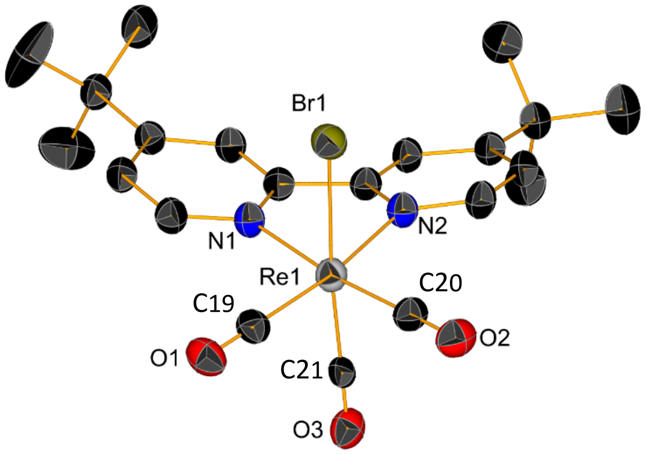

Table S4d′ Selected Bond Angles for **6**.

Atom	Atom	Atom	Angle/°	Atom	Atom	Atom	Angle/°	Atom	Atom	Atom	Angle/°
N1	Re1	Br1	86.09(12)	N1	Re1	N2	73.84(16)	C20	Re1	C19	88.0(2)
N2	Re1	Br1	84.23(12)	C19	Re1	Br1	95.54(17)	C20	Re1	C21	89.6(2)
C19	Re1	N1	96.6(2)	C19	Re1	N2	170.5(2)	C21	Re1	Br1	174.51(15)
C19	Re1	C21	89.9(2)	C20	Re1	Br1	89.84(17)	C21	Re1	N2	90.5(2)
C20	Re1	N1	174.1(2)	C20	Re1	N2	101.5(2)	C21	Re1	N1	94.09(19)

Table S4e Selected Bond Lengths for **7**.

Atom	Atom	Length/Å
Re1	Br1	2.605(2)
Re1	N1	2.145(17)
Re1	N2	2.148(15)
Re1	C13	1.93(2)
Re1	C14	1.917(18)
Re1	C15	2.01(2)
O1	C13	1.12(2)
O2	C14	1.15(2)
O3	C15	1.03(2)

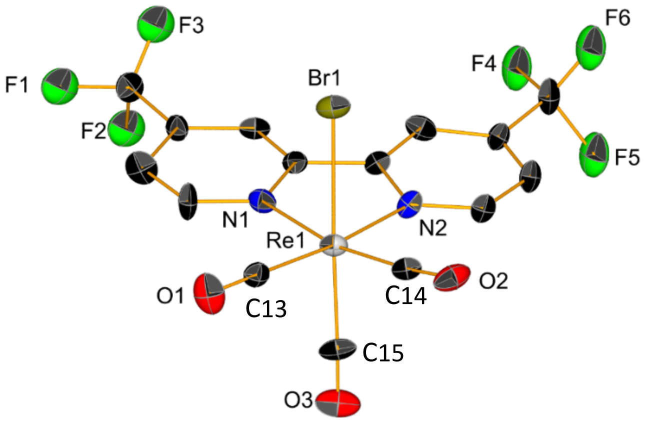

Table S4e′ Selected Bond Angles for **7**.

Atom	Atom	Atom	Angle/°	Atom	Atom	Atom	Angle/°	Atom	Atom	Atom	Angle/°
N1	Re1	Br1	83.2(4)	N1	Re1	N2	75.6(6)	C14	Re1	C13	88.5(8)
N2	Re1	Br1	84.7(4)	C13	Re1	Br1	92.8(6)	C14	Re1	C15	92.2(8)
C13	Re1	N1	97.8(7)	C13	Re1	N2	173.1(7)	C15	Re1	Br1	176.2(6)
C13	Re1	C15	90.9(8)	C14	Re1	Br1	89.0(5)	C15	Re1	N1	95.3(7)
C14	Re1	N1	170.2(7)	C14	Re1	N2	97.8(7)	C15	Re1	N2	91.6(7)

Table S4f Selected Bond Lengths for **8**.

Atom	Atom	Length/Å
Re1	Br1	2.6307(6)
Re1	N1	2.162(3)
Re1	N1[1]	2.162(3)
Re1	C10	1.912(5)
Re1	C10[1]	1.912(5)
Re1	C11	1.940(7)
O1	C10	1.158(6)
O2	C11	1.074(7)

[1]+X,+Y,3/2-Z

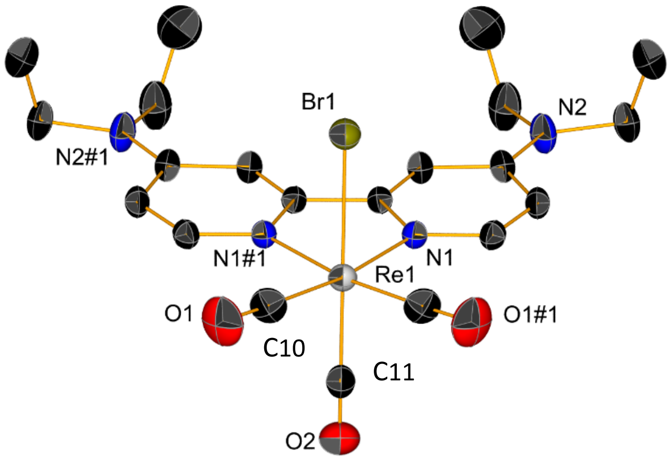

Table S4f′ Selected Bond Angles for **8**.

Atom	Atom	Atom	Angle/°	Atom	Atom	Atom	Angle/°	Atom	Atom	Atom	Angle/°
N1[1]	Re1	Br1	84.59(8)	N1	Re1	Br1	84.59(8)	C10[1]	Re1	C11	90.7(2)
N1	Re1	N1[1]	74.49(15)	C10[1]	Re1	Br1	91.37(16)	C10	Re1	C11	90.7(2)
C10	Re1	Br1	91.37(16)	C10	Re1	N1[1]	98.41(17)	C11	Re1	Br1	177.06(17)
C10[1]	Re1	N1[1]	172.10(16)	C10[1]	Re1	N1	98.41(17)	C11	Re1	N1	93.08(16)
C10	Re1	N1	172.10(16)	C10	Re1	C10[1]	88.4(3)	C11	Re1	N1[1]	93.08(16)

[1]+X,+Y,3/2-Z

Table S4g Selected Bond Lengths for **10**.

Atom	Atom	Length/Å
Re1	Br1	2.6162(7)
Re1	Br2A	2.473(15)
Re1	N1	2.158(5)
Re1	N2	2.171(4)
Re1	C25	1.904(6)
Re1	C26	1.934(6)
Re1	C27	1.885(13)
O1	C25	1.164(8)
O2	C26	1.148(7)
O3	C27	1.189(19)

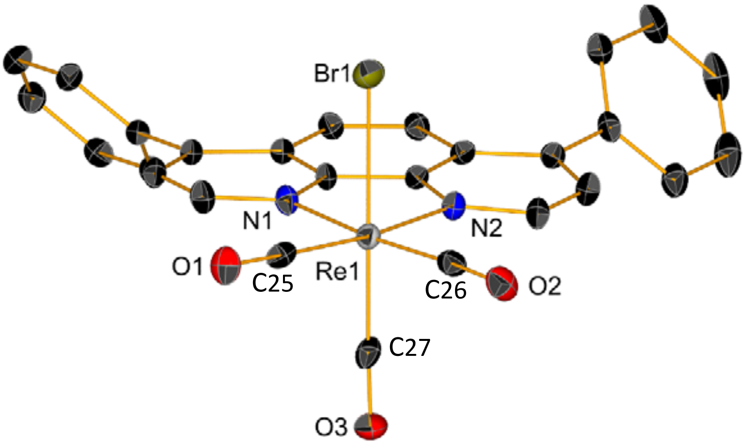

Table S4g′ Selected Bond Angles for **10**.

Atom	Atom	Atom	Angle/°	Atom	Atom	Atom	Angle/°	Atom	Atom	Atom	Angle/°
N1	Re1	Br1	83.46(14)	C25	Re1	N2	171.8(2)	C25	Re1	C26	89.4(2)
N1	Re1	N2	75.19(17)	C26	Re1	Br1	94.49(17)	C27	Re1	C25	89.8(3)
N2	Re1	Br1	85.69(12)	C26	Re1	N2	98.4(2)	C26	Re1	N1	173.4(2)
C25	Re1	Br1	91.32(17)	C27	Re1	N2	93.1(3)	C27	Re1	N1	95.3(3)
C25	Re1	N1	96.9(2)	C27	Re1	C26	86.6(3)	C27	Re1	Br1	178.4(3)

Table S4h Selected Bond Lengths for ***cis*-11**.

Atom	Atom	Length/Å
Re1	Br1	2.5533(8)
Re1	Br3	2.466(12)
Re1	N1	2.159(5)
Re1	N2	2.163(5)
Re1	C25	1.973(7)
Re1	C27	1.968(18)
O1	C25	1.063(8)
O3	C27	1.092(18)

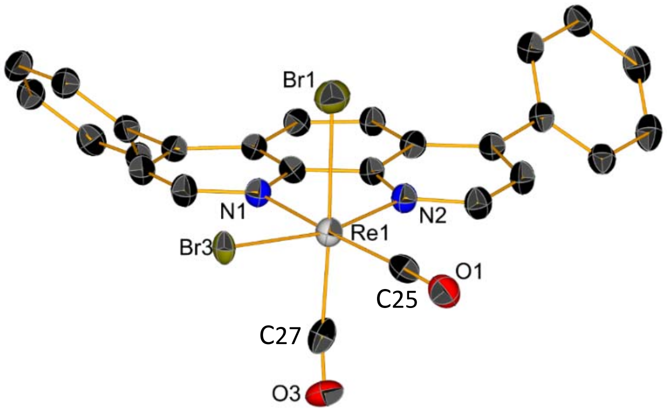

Table S4h′ Selected Bond Angles for ***cis*-11**.

Atom	Atom	Atom	Angle/°	Atom	Atom	Atom	Angle/°	Atom	Atom	Atom	Angle/°
Br3	Re1	Br1	86.5(4)	N1	Re1	Br1	85.24(13)	C27	Re1	N1	93.0(6)
N1	Re1	N2	75.15(17)	N1	Re1	Br3	92.6(3)	C27	Re1	Br1	177.5(6)
N2	Re1	Br1	86.15(13)	N2	Re1	Br3	166.2(4)	C27	Re1	C25	85.5(6)
C25	Re1	Br3	94.7(4)	C25	Re1	Br1	96.45(18)	C27	Re1	N2	95.1(6)
C25	Re1	N2	97.7(2)	C25	Re1	N1	172.6(2)				

Table S4i Selected Bond Lengths for ***mer*-12**.

Atom	Atom	Length/Å
Re1	Br1	2.5533(8)
Re1	Br2	2.479(3)
Re1	Br3	2.466(12)
Re1	N1	2.159(5)
Re1	N2	2.163(5)
Re1	C25	1.973(7)
O1	C25	1.063(8)

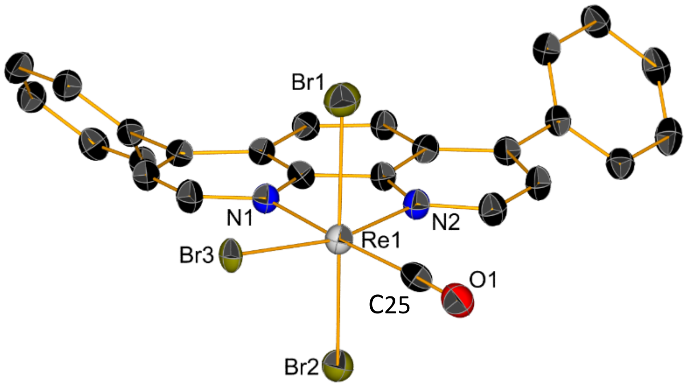

Table S4i′ Selected Bond Angles for ***mer*-12**.

Atom	Atom	Atom	Angle/°
Br2	Re1	Br1	170.34(6)
N1	Re1	Br1	85.24(13)
N1	Re1	Br3	92.6(3)
N2	Re1	Br1	86.15(13)
N2	Re1	Br3	166.2(4)
Br3	Re1	Br1	86.5(4)
N1	Re1	Br2	87.41(14)
N1	Re1	N2	75.15(17)
N2	Re1	Br2	85.92(14)
C25	Re1	Br3	94.7(4)
C25	Re1	Br1	96.45(18)
C25	Re1	N2	97.7(2)
C25	Re1	Br2	90.07(19)
C25	Re1	N1	172.6(2)

References

1. Richter, S.G.; Elli, D.; Kim, H.K.; Hendrickx, A.P.; Sorg, J.A.; Schneewind, O.; Missiakas, D. Small molecule inhibitor of lipoteichoic acid synthesis is an antibiotic for Gram-positive bacteria. *Proc. Natl. Acad. Sci. USA* **2013**, *110*, 3531-3536, doi:10.1073/pnas.1217337110.
2. Craney, A.; Romesberg, F.E. The inhibition of type I bacterial signal peptidase: Biological consequences and therapeutic potential. *Bioorg. Med. Chem. Lett.* **2015**, *25*, 4761-4766, doi:10.1016/j.bmcl.2015.07.072.
3. Olatunji, S.; Yu, X.; Bailey, J.; Huang, C.Y.; Zapotoczna, M.; Bowen, K.; Remskar, M.; Muller, R.; Scanlan, E.M.; Geoghegan, J.A., et al. Structures of lipoprotein signal peptidase II from Staphylococcus aureus complexed with antibiotics globomycin and myxovirescin. *Nat. Commun.* **2020**, *11*, 140, doi:10.1038/s41467-019-13724-y.
4. Hebditch, M.; Warwicker, J. Web-based display of protein surface and pH-dependent properties for assessing the developability of biotherapeutics. *Sci. Rep.* **2019**, *9*, 1969, doi:10.1038/s41598-018-36950-8.
5. Warwicker, J. Continuum dielectric modelling of the protein-solvent system, and calculation of the long-range electrostatic field of the enzyme phosphoglycerate mutase. *J. Theor. Biol.* **1986**, *121*, 199-210, doi:10.1016/s0022-5193(86)80093-5.
